# Functional dissection and assembly of a small, newly evolved, W chromosome-specific genomic region of the African clawed frog *Xenopus laevis*

**DOI:** 10.1371/journal.pgen.1010990

**Published:** 2023-10-04

**Authors:** Caroline M. S. Cauret, Danielle C. Jordan, Lindsey M. Kukoly, Sarah R. Burton, Emmanuela U. Anele, Jacek M. Kwiecien, Marie-Theres Gansauge, Sinthu Senthillmohan, Eli Greenbaum, Matthias Meyer, Marko E. Horb, Ben J. Evans

**Affiliations:** 1 Biology Department, McMaster University, Hamilton, Ontario, Canada; 2 Department of Botany and Plant Pathology, Oregon State University, Corvallis, Oregon, United States of America; 3 Eugene Bell Center for Regenerative Biology and Tissue Engineering and National Xenopus Resource, Marine Biological Laboratory, Woods Hole, Massachusetts United States of America; 4 The School of Biological Sciences, University of Aberdeen, Aberdeen, United Kingdom; 5 Department Zoology, Ahmadu Bello University, Zaria, Nigeria; 6 Department of Pathology and Molecular Medicine, McMaster University, Hamilton, Ontario, Canada; 7 Department of Evolutionary Genetics, Max Planck Institute for Evolutionary Anthropology, Leipzig, Germany; 8 Department of Biological Sciences, The University of Texas at El Paso, El Paso, Texas, United States of America; Duke University Medical Center, UNITED STATES

## Abstract

Genetic triggers for sex determination are frequently co-inherited with other linked genes that may also influence one or more sex-specific phenotypes. To better understand how sex-limited regions evolve and function, we studied a small W chromosome-specific region of the frog *Xenopus laevis* that contains only three genes (*dm-w*, *scan-w*, *ccdc69-w*) and that drives female differentiation. Using gene editing, we found that the sex-determining function of this region requires *dm-w* but that *scan-w* and *ccdc69-w* are not essential for viability, female development, or fertility. Analysis of mesonephros+gonad transcriptomes during sexual differentiation illustrates masculinization of the *dm-w* knockout transcriptome, and identifies mostly non-overlapping sets of differentially expressed genes in separate knockout lines for each of these three W-specific gene compared to wildtype sisters. Capture sequencing of almost all *Xenopus* species and PCR surveys indicate that the female-determining function of *dm-w* is present in only a subset of species that carry this gene. These findings map out a dynamic evolutionary history of a newly evolved W chromosome-specific genomic region, whose components have distinctive functions that frequently degraded during *Xenopus* diversification, and evidence the evolutionary consequences of recombination suppression.

## Author summary

In species with separate male and female individuals, sexual differentiation is sometimes triggered by genes or genetic variation that are found only in one sex. Interestingly however, not all sex-specific genes are necessarily important for sexual differentiation, viability, or fertility. We studied functional evolution of a small female-specific region on the W chromosome of the frog *Xenopus laevis* by using genome editing to disable function independently in each of the three genes in this region and capture sequencing in multiple species to explore how this genomic region was assembled and modified by evolution. Consistent with previous studies, knockout of *dm-w* caused complete female to male sex reversal, including masculinization of genes expressed in the developing gonad. Disabling other two female specific genes (*scan-w*, *ccdc69-w*), however, had no discernable effect on female viability, differentiation, or fertility. During radiation of the genus of *Xenopus*, genomic components of the W chromosome-specific region found in *X*. *laevis* coalesced in an ancestor but degraded or were lost in several other *Xenopus* descendant species. Together, these findings demonstrate functional independence and non-essentiality of three W chromosome-specific genes and document extensive among-species genetic variation that is in line with expectations for genomic regions that lack genetic recombination.

## Introduction

Proteins with functional associations are sometimes encoded by genes that are genetically linked in the genome [1] or in the same physical space in the nucleus [2], which may promote their co-regulation. Groups of tightly linked genes are thought to orchestrate many complex phenotypes [3] such as behaviour [4], mimicry [5], color [6], heterostyly [7], male reproductive behaviour [8], offspring sex ratio [9], and (perhaps most notably) sexual differentiation [10]. Genetic associations between alleles of different loci can be favored under several scenarios such as heterogeneity of environmental conditions (if certain combinations of alleles are beneficial in some habitats but not others) or negative epistasis [if certain combinations of alleles are deleterious; 11]. Recombination arrest could be favored by natural selection to maintain advantageous combinations of alleles across multiple genes [12–16] and mechanistically could be achieved by genomic changes such as inversions or allelic divergence. Expansion of recombination suppression could be triggered by regulatory changes [17,18], sexual antagonism [15,19], heterozygote advantage and balancing selection [20,21], meiotic drive [19], and neutral processes [22,23].

Because recombination suppression causes co-inheritance of genes that are physically linked to the sex-determining locus, multiple genes within sex-specific portions of sex chromosomes may act together to sculpt sex-specific phenotypes [10]. However, in some cases, sex-linked genes encode diverse phenotypes, including some that are not directly related to sex determination. For example, the male-specific portion of the human Y-chromosome encodes a protein (*Sry*) that triggers male primary gonadal differentiation, and also several other genes that function long after primary sexual differentiation has been achieved [albeit related to male fertility; 24].

In principle, different genes in a linked region could have epistatic interactions that influence one phenotype [25]. If this were the case, each gene would be necessary but not individually sufficient to produce the phenotype that is controlled by the linked region, or multiple components within this region could have modifier effects on this phenotype. In the case of a sex-determining region, for example, sexual differentiation might require a functional version of all genes in the region. In some plants, for example, male differentiation is orchestrated by two genes; natural selection may have favoured the co-localization of both on a male-specific region in kiwis [26,27]. At the other extreme is the possibility that individual genes on a sex-determining region lack strong epistatic interactions, with each locus influencing a different phenotype. For example, one locus could influence primary (gonadal) sexual differentiation, and another could influence secondary (non-gonadal) differentiation, or even a non-essential or subtle trait. Because they occur in only one sex, each gene in a sex-specific genomic region necessarily must have sex-specific phenotypic influences. Clearly, however, not all loci on a sex-specific region are necessarily required for the most fundamental aspects of sexual differentiation, which include viability and reproduction.

### A small W chromosome-specific genomic region in the African clawed frog (*Xenopus laevis*)

To explore how sex-limited genomic regions arise, function, and change over time, we studied a small female-determining genomic region on the W chromosome of the African clawed frog, *Xenopus laevis*. This region is ~278 kilobases (kb) long, located on chromosome 2L, and contains only three W chromosome-specific genes [28]: *dm-w*, *scan-w*, and *ccdc69-w*. No gametolog of these three W chromosome-specific genes is known to be present on the Z chromosome. Most or all of this W-specific region is not found on the Z chromosome, probably because this region formed from multiple insertion events into the W chromosome that were not shared with the Z chromosome. Low sequence homology between the W chromosome-specific region and the Z chromosome [apart from repetitive elements; 28] presumably contributes to recombination suppression in this region.

There are strong reasons to suspect that sex determination in *X*. *laevis* is triggered by the presence or absence of this W chromosome-specific genomic region, as opposed to environmental factors, or a polygenic trigger that involves genes outside of this W chromosome-specific region (such as the sex-related genes *dmrt1L* and *dmrt1S* which reside on chromosomes 1L and 1S, respectively). One of these genes in particular–*dm-w*–is thought to be the main trigger for primary (gonadal) sexual differentiation of female *X*. *laevis* [29, 30]. In a survey of 24 females and 12 males in nature, all females and no males carried *dm-w* [31]. Female-specificity of *dm-w* was also suggested in *X*. *gilli* based on a survey of 13 females and seven males [31]. In a laboratory-reared family that included 17 daughters and 20 sons, reduced representation genome sequencing recovered a strong association with phenotypic sex exclusively on the region of Chromosome 2L that contains the W chromosome-specific region [32]. In three of nine or three of seven transgenic (ZZ) males (depending on the construct used), insertion of *dm-w* by restriction enzyme-mediated integration resulted in the development of ovotestes, which contain both ovarian and testicular structures [29]. In the transgenic males that did not develop ovotestis, testis tissue developed, but the *dm-w* transgene was generally lowly expressed [29]. In three of 11 (ZW) female tadpoles and 10 of 38 female (ZW) adults that carried an RNA interference transgene against *dm-w*, abnormal gonads developed that were partially sex-reversed [29,30] and gonads of two of 38 female (ZW) adults that were transgenic for a *dm-w* knockdown construct were fully sex reversed [30]. Other genetically female (ZW) individuals with and an RNA interference transgene developed into phenotypic females [29,30]. The variable effects of *dm-w* transgenes in genetic (ZZ) males and *dm-w* inactivation transgenes in genetic (ZW) females could indicate that dosages of other W-linked genes or Z-linked loci also influence sexual differentiation, or alternatively this could have a methodological basis (e.g., positional effects of the *dm-w* transgene or incomplete inactivation of *dm-w* by RNA interference).

However, *dm-w* is not the trigger for female-differentiation in at least some other *Xenopus* species (apart from *X*. *laevis* and *X*. *gilli*) that carry this gene. PCR surveys suggest that all individuals of both sexes of the octoploid species *X*. *itombwensis* carry *dm-w* (it was present in all five females and all 20 males surveyed) [31]. Additionally, *dm-w* is not female-specific in *X*. *clivii* (it was present in five of 29 males surveyed), *X*. *pygmaeus* (it was present in two of 11 males), or *X*. *victorianus* (it was present in five of 15 males) [31] and it is probably not required for female differentiation in two of these species (it was not detected in five of 16 *X*. *clivii* females and three of nine *X*. *pygmaeus* females [31]).

In adult *X*. *laevis*, the other two W chromosome-specific genes in *X*. *laevis–scan-w* and *ccdc69-w–*have substantial expression levels in either the brain and stomach or the gonads and brain respectively [28]. In tadpoles, *scan-w* and *ccdc69-w* are both expressed in the developing gonads during and after sexual differentiation [28]. The scan domain, which is present in the *Scan-w* protein [28], is a highly conserved motif that facilitates dimerization and is typically found near the N-terminus of vertebrate C_2_H_2_ zinc-finger proteins, but most of these proteins have unknown function [33]. The *Ccdc69* protein, which is paralogous to *ccdc69-w*, is involved with microtubule binding activity and spindle formation during cytokinesis [34].

That their closest paralogs in the autosomes are not tightly linked [28,29,35–37] suggests that these three loci each arose by independent duplication events that inserted them in juxtaposition in the ancestral genomic region that became the W chromosome-specific portion of the *X*. *laevis* genome. These duplication events are separate from and subsequent to those associated with allotetraploidization in *Xenopus* (which occurred at least two separate times to generate the ancestors of extant allotetraploid species) [38,39]. These allotetraploid species (ancestral and extant) have two subgenomes that are respectively derived from two different diploid ancestors. The subgenomes of the most recent common allotetraploid ancestor of *X*. *laevis* and *X*. *clivii* are denoted “L” and “S” [40] and homeologous genes in each subgenome generally include these letters as a suffix (e.g., *dmrt1L* and *dmrt1S* are homeologs that by definition are duplicated genes that arose from genome duplication). Strikingly, *dm-w* appears to be a chimerical gene, whose components are derived from as many as three different sources including: (i) the second and third exons and flanking regions, which formed from gene duplication of *dmrt1S* [28,35,36], (ii) the fourth exon and flanking regions, which arose from a noncoding DNA transposon called hAT-10 [36], and (iii) the first exon and flanking regions, which does not have discernible homology to *dmrt1S*, is rich in transposable elements, and has unclear origins [41]. A recent genome assembly for *X*. *laevis* (version 10.1) suggests that the transcribed regions of *dm-w* and *scan-w* overlap because exons 4–6 of *scan-w* are located in the first intron of *dm-w*. All three of these genes are transcribed in the same direction, which is in the reverse orientation of the coordinates for chromosome 2L in the *X*. *laevis* genome assembly. Combined with the differing genomic locations of paralogous genes [28], the overlapping transcribed regions of *dm-w* and *scan-w* is consistent with a chimerical origin of *dm-w* wherein exons 2 and 3 originated via separate duplication/translocation events from exon 1 and exon 4 [29,36,41].

We set out to better understand evolution and function of the W-linked sex-linked genomic region of *X*. *laevis*. We explored function of each of the three genes in this region by independently inactivating each one of them using CRISPR/Cas9 gene editing, and we then explored their mutant phenotypes in terms of sex-determination, fertility, and gonadal transcriptomics. We also investigated the evolutionary histories of each of these three genes using targeted capture sequencing across almost all *Xenopus* species and PCR assays, with interpretations in a phylogenetic context. These efforts provide comprehensive insights into functional evolution and assembly of a small W chromosome-specific sex-determining region, demonstrate non-overlapping and partially non-essential activities of its components, and evidence functional degeneration of each component–findings that are in step with the expectation that the efficacy of natural selection is reduced in genomic regions lacking recombination [42,43].

## Results

### Female differentiation of *X*. *laevis* is triggered by *dm-w*, but not *scan-w* or *ccdc69-w*

To further characterize their functional roles, we created a knockout line for each of three genes: *dm-w*, *scan-w* and *ccdc69-w* in *X*. *laevis* using CRISPR/Cas9 (S1 Text and S1 Fig). F0 mosaic individuals were crossed with wildtype individuals to generate non-mosaic (i.e., containing only the mutant allele in all cells) F1 individuals. For each knockout line, viable F1 individuals were recovered, which demonstrates non-essentiality for each of these genes for viability of genetic females. Fertility of F1 knockout individuals was assessed by crossing them to wildtype individuals with the opposite sex phenotype; gonadal gross anatomy and histology of F1 individuals were then characterized after euthanasia.

**Fig 1:**
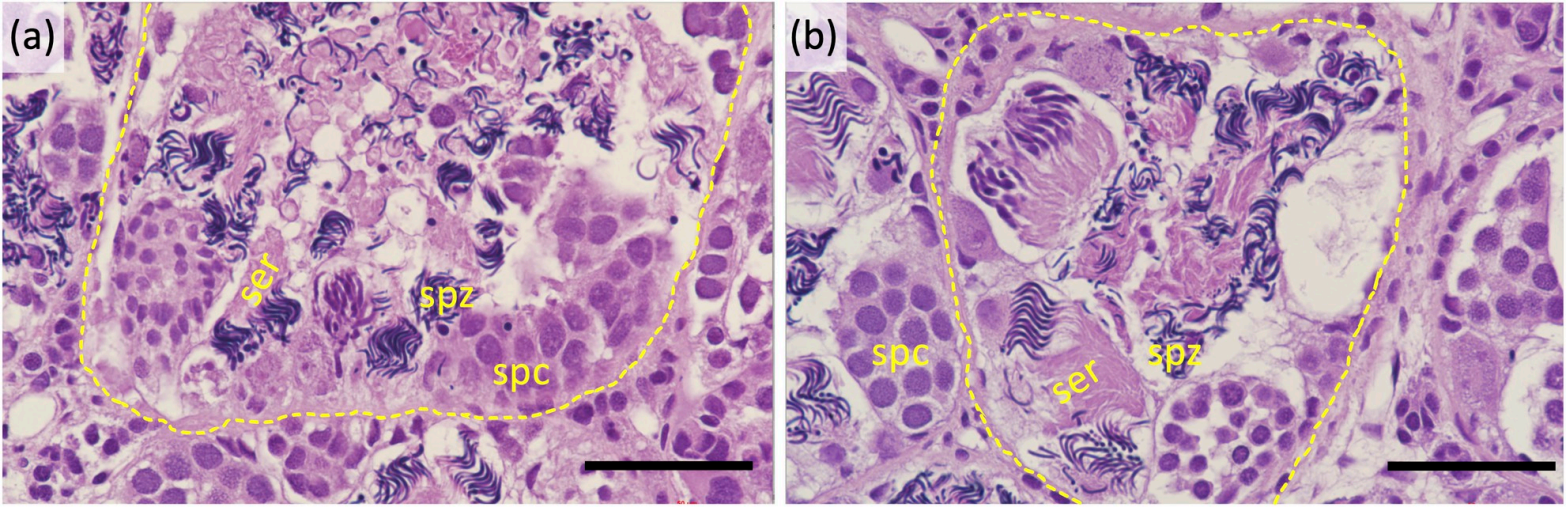
Testis histology of (a) a wildtype male and (b) a sex reversed F1 female carrying a *dm-w* knockout mutation. Black bars are 50 μm; individuals’ identification numbers are (a) 17FO and (b) 1847. Dotted circles indicate the margins of seminiferous tubules, and Sertoli cells (ser), spermatocytes (spc) and spermatozoa (spz) are labeled.

In the F0 and F1 generations, genetic females carrying the *dm-w* knockout mutation (a 10 bp deletion that was confirmed by Sanger sequencing; S1 Fig) developed into phenotypic males. When F0 individuals were crossed with wildtype (ZW) females, viable F1 offspring were produced, which demonstrates that the sex reversed F0 females developed into phenotypically fertile males. In the F1 generation, a wildtype (ZW) female and a phenotypically male (ZW*) mutant female (where W* indicates the W chromosome carrying an inactivated copy of *dm-w* that was confirmed by Sanger sequencing) were crossed to produce offspring with four different sex chromosome phenotypes: W*Z (n = 6), W*W (n = 8), WZ (n = 5), and ZZ (n = 6). All W*Z individuals developed into phenotypic males and all W*W individuals developed into phenotypic females; wildtype offspring matched their expected sexes with WZ individuals developing into phenotypic females and ZZ individuals developing into phenotypic males. Fertility of a W*W female was confirmed by a cross to a phenotypically male (ZW*) mutant female. This cross produced offspring that were WZ (n = 8), W*W (n = 16), and W*Z or W*W* (n = 19 in total for these two offspring genotypes; we did not distinguish them because their *dm-w* sequences are identical for the hemizygous mutant allele and the homozygous mutant allele). As expected, the W*Z or W*W* offspring were phenotypically male and the W*W and WZ offspring were phenotypically female. Histological analysis of testis tissue from four F2 sex-reversed *dm-w* mutant females (W*Z) is consistent with complete sex reversal, including normal sperm development (Figs 1 and S2). We also were able to obtain offspring from a sex-reversed genetic female and a wildtype female using natural mating after both individuals were injected with human chorionic gonadotropin (which is generally required to elicit sexual behavior in captive *Xenopus*). This indicates that, in addition to producing normal sperm and being fertile, sex-revered genetic females also exhibit sexual behaviour of phenotypic males (amplexus).

Together these results indicate in *X*. *laevis* that (i) loss of function mutation in *dm-w* causes complete sex reversal of a genetic female to a fertile male, (ii) *dm-w* is not necessary for viability of genetic females which develop into phenotypic males, and (iii) having a functional copy of *scan-w* and *ccdc69-w* does not prevent development of the male phenotype by genetic females that carry a knockout mutation for *dm-w*.

All F1 *scan-w* knockout individuals (n = 10 individuals with 20 bp deletion that creates a premature stop codon; S1 Fig) and all *ccdc69-w* knockout individuals (n = 9 individuals in total including two with a 22 bp deletion creates a premature strop codon, and seven with a 214 bp deletion associated with a 12 bp insertion that also creates a premature strop codon, S1 Fig) developed into phenotypically normal (and gravid) adult females. These observations demonstrate that neither *scan-w* nor *ccdc69-w* is required for female differentiation. When crossed to wildtype males, F1 *scan-w* and *ccdc69-w* knockout lines each produced viable F2 individuals, demonstrating that *scan-w* and *ccdc69-w* are not required for female fertility.

### Variable transcriptomic responses to knockout of different W-specific genes

In females, *dm-w* is expressed in the developing gonad during sexual differentiation, and in adult ovary and liver [28,44]. Using RNAseq data, we confirmed female-specificity of *dm-w* in the developing mesonephros+gonad (S3 Fig). Because *scan-w* and *ccdc69-w* were not present in the most recent reference transcriptome (version 10), in order to evaluate expression of these loci we added previously reported transcripts from [28] to this reference transcriptome and performed a separate quantification and normalization. Both genes were found to have zero or almost zero expression in the tadpole stage 50 mesonephros+gonad of all individuals, whether male or female, knockout or wildtype. While this does not rule out expression in other tissues or developmental stages, it is at odds with real-time PCR results reported previously that detected expression of these genes in female tadpole stage 50 mesonephros+gonad tissue [28].

**Fig 2:**
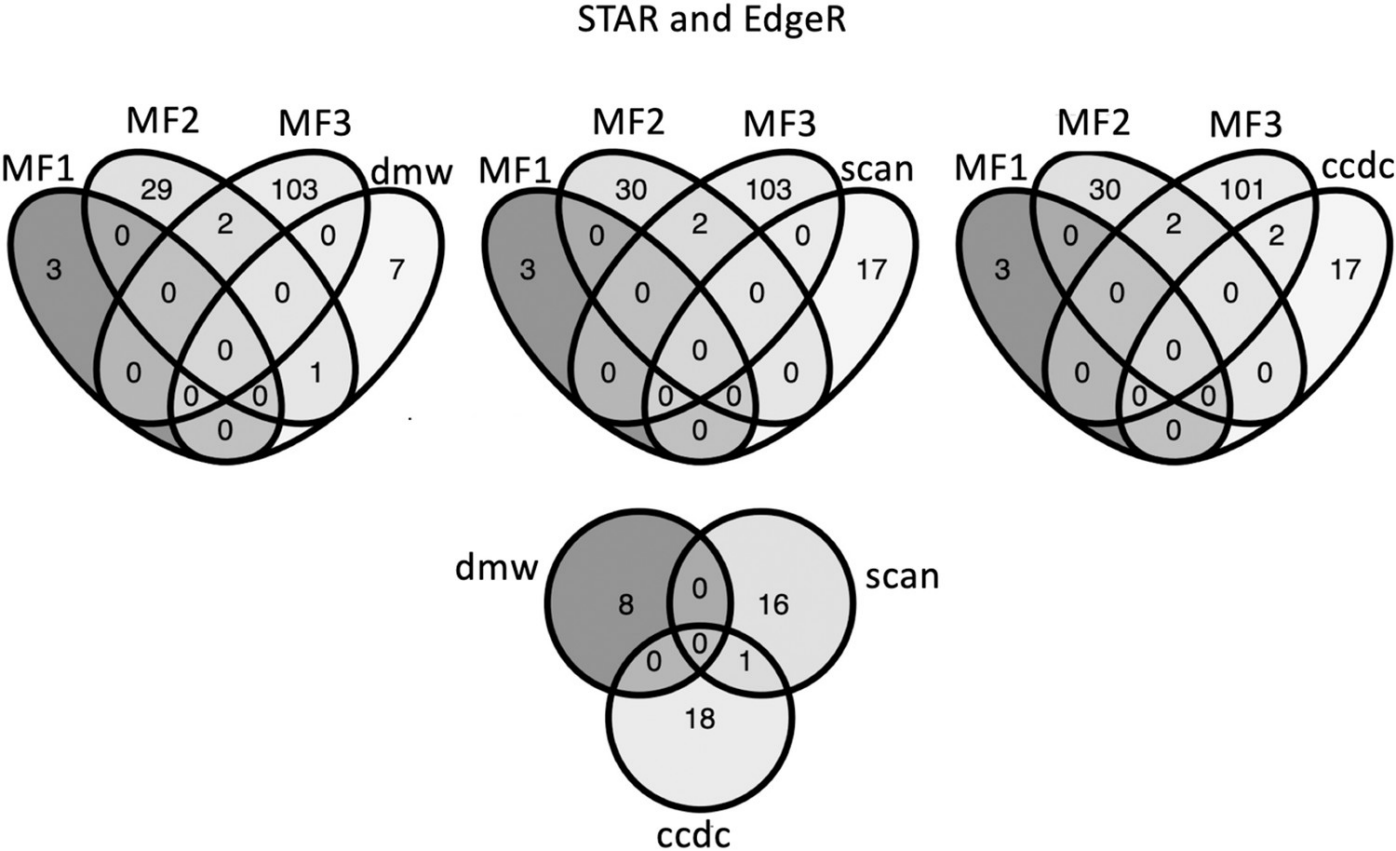
Venn diagrams showing the numbers of overlapping and batch-specific differentially expressed genes in three batches where sex-specific expression was considered (MF1, MF2, MF3) and knockout to wildtype comparison for each knockout line: *dm-w* (dmw), *scan-w* (scan), and *ccdc69-w* (ccdc). Results are shown for quantification using STAR and analysis of differential expression using EdgeR. In the analyses of sex-specific expression, female expression is the reference; in the analysis of knockout expression, wildtype (female) expression is the reference.

We then compared expression of genes in the developing mesonephros+gonad of genetically female knockout and wildtype individuals at tadpole stage 50. Irrespective of the methods for transcript quantification or analysis of differential expression (Methods), the sets of differentially expressed genes for each mutant line (mutant versus wildtype sisters; S1 Table) were almost entirely non-overlapping with each other or with three independent analyses of sex-biased expression in wildtype individuals (wildtype brothers versus wildtype sisters; S1 Table and Figs 2 and S4–S6). These results may be attributable in part to batch effects discussed below, but are also consistent with the distinctive functions of each of these genes that are evidenced respectively by the adult knockout phenotypes (sex-reversal for *dm-w* but not for *scan-w* or *ccdc69-w*).

Analysis of differential expression of the *dm-w* knockout line compared to wildtype siblings found 8–33 significantly differentially expressed genes depending on the analysis pipeline (S1 Table and Figs 2 and S4–S6). Gene ontology of differentially expressed genes in the *dm-w* knockout line did not recover significant enrichments in biological process, molecular function, or cellular component in any analysis pipeline (S2 Table).

Analysis of differential expression of the *scan-w* knockout line identified between 17 and 34 significantly differentially expressed genes, depending on the analysis pipeline (S1 Table and Figs 2 and S4–S6). Gene ontology of differentially expressed genes identified enrichments in cellular components associated with extracellular space for results from some analysis pipelines (Kallisto + DeSeq2, STAR + DeSeq2; S2 Table).

Analysis of differential expression of the *ccdc69-w* knockout line identified 17–263 significantly differentially expressed genes, depending on the analysis pipeline (S1 Table and Figs 2 and S4–S6). Gene ontology of differentially expressed genes in the *ccdc69-w* knockout line recovered a significant enrichment of genes involved in biological processes such as oxygen transport, detoxification, molecular functions such as binding of oxygen and heme, and cellular components associated with hemoglobin (S2 Table).

We also evaluated sex-biased expression in the developing mesonephros+gonad in wildtype individuals. Here again, significantly differentially expressed genes were generally non-overlapping across these three independent clutches (MF1, MF2, MF3), even though the genotypes in each treatment were the same (i.e., wildtype male versus wildtype female). Overall, we found substantial among-batch variation in the number and identity of transcripts with significant sex-biased expression in three different batches of wildtype female and male mesonephros +gonad transcriptomes (Figs 2 and S4–S6). Gene ontology analysis identified an enrichment in biological processes including oxygen transportation and hydrogen peroxide catabolism, molecular functions such as haptoglobin and iron binding and oxygen carrier activity, and cellular components such as the hemoglobin complex (S2 Table).

The batch effects in the three wildtype analyses (MF1, MF2, MF3) could be in part due to technical differences, such as among-batch variation in the number of biological replicates and the number of reads per individual. It could also stem from among-batch developmental asynchrony in the timing of gonadal differentiation versus the morphological features that demarcate tadpole stage 50. Transcriptomic variation could also stem from among-individual genetic variation (e.g., nucleotide and epigenetic variation, maternal proteins); and variation among batches could be attributable to minor differences between tanks in temperature and other environmental parameters.

### Masculinization of the developing gonad transcriptome in the *dm-w* knockout

The comparison between the *dm-w* knockout and wildtype transcriptomes discussed above did not recover a large number of shared significantly differentially expressed transcripts, and those that were recovered did not have a significant enrichment for sex-related functional ontologies. In addition to batch effects and technical variation, the inclusion of mesonephros tissue–which are substantially (>20X) larger than the gonads at tadpole stage 50 –in our transcriptomic analyses may have decreased the signal of sex-biased expression in the gonad transcriptomes.

**Fig 3:**
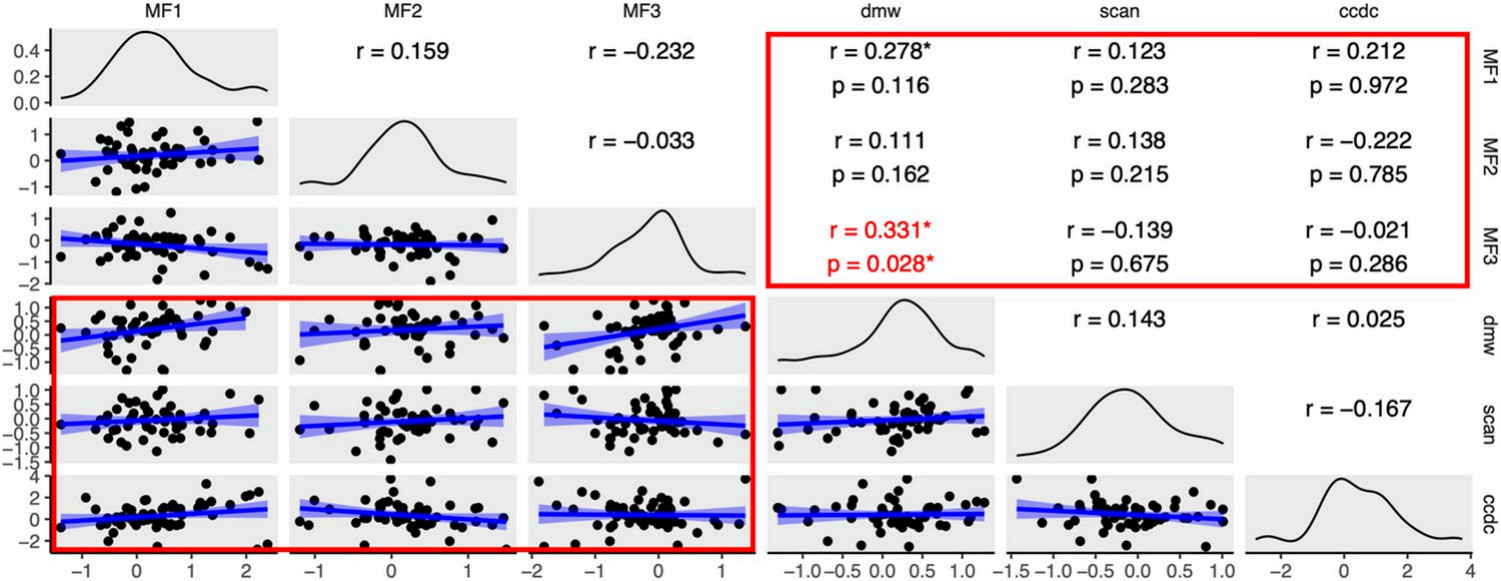
Analysis of transcriptome masculinization using the STAR-EdgeR pipeline. Pairwise correlations between non-outlier log2 fold changes of sex-related genes are plotted below the diagonal. Pearson’s correlation coefficients are plotted above the diagonal with asterisks indicating significantly positive correlation coefficients. The diagonal is a density plot of log2 fold changes for each analysis. For pairwise comparisons between wildtype analyses (MF1, MF2, MF3) and the knockout and wildtype analysis (dmw, scan, ccdc), which are highlighted by red boxes, p-values of permutation tests are reported in the top below each correlation coefficient, with red font and a red asterisk highlighting significantly positive correlations based on permutation tests.

However, it is still possible that knockout of *dm-w* did lead to masculinization of the transcriptome of the mesonephros+gonad complex at this early stage of sexual differentiation, but that we lacked statistical power to detect this. To explore this possibility, we focused on 74 sex-related genes (S3 Table) and tested whether the knockout:wildtype expression ratios of these genes were positively correlated with the wildtype male:female expression ratios of these genes at the same developmental stage and tissue type. For three of four analysis pipelines, there was a significantly positive correlation between the expression ratios from the *dm-w* knockout:wildtype female comparison and the expression ratios for the same genes in the MF3 wildtype male:wildtype female comparison (Figs 3 and S7–S8); this correlation was positive in the fourth pipeline but not significantly so (S9 Fig). Permutation tests indicated that these correlations were significantly more positive than expected by chance for three of four analysis pipelines (all except Kallisto-EdgeR, S9 Fig). There was also a significantly positive correlation between the expression ratios the *dm-w* knockout:wildtype female comparison and the expression ratios for the same genes in the MF1 wildtype male:wildtype female comparison for three analysis pipelines (Figs 3, S7 and S9), but permutation tests indicate that none of these correlations is significantly more positive than expected by chance. Overall, these results indicate that the *dm-w* knockout transcriptomes are masculinized compared to wildtype females.

A few other correlations were significantly positive (e.g., between the *scanw-w* knockout analysis and the MF2 analysis for one of the four pipelines, and between the *ccdc69-w* knockout analysis and the MF1 analysis for two pipelines or the MF3 analyses for one pipeline). However, permutation tests indicate that only the first of these comparisons (between the *scanw-w* knockout analysis and the MF2 analysis) is significantly more positive than expected by chance (Kallisto-EdgeR, S9 Fig). We expected expression ratios to generally be positively correlated between the *ccdc69-w* knockout analysis and the MF1 analysis because the wildtype females in these analyses were the same. Taken together, these results indicate that there is no evidence for masculinization of the transcriptomes of the *ccdc69-w* knockout lines, and that evidence for masculinization of the *scan-w* knockout lines is modest.

### Assembly of the W chromosome-specific portion of the *X*. *laevis* genome

The components of *dm-w* were assembled during diversification of *Xenopus* [31,35,36,41] around 20 million or more years ago [37,38,45,46,47]. To further explore the origins of genetic components of the W chromosome-specific region of the *X*. *laevis* W chromosome, we collected capture sequence data for exon 4 of *dm-w*, exons 4 and 5 of *scan-w*, and both exons of *ccdc69-w* in the same sample of *Xenopus* species as previously [S4 Table; 31]. This included all *Xenopus* species except *X*. *fraseri*, and almost all individuals from each species were female. Capture sequencing of *dm-w* exons 2 and 3 from the same samples were previously reported [31]. Exon 1 of *dm-w* is small and non-coding and was not intentionally targeted for capture sequencing. However, as detailed below, *dm-w* exon 1 was sequenced as “by-catch” of *scan-w* exon 4 in some species. *Scan-w* has six exons but we focused our attention on only exons 4 and 5 because the other exons are highly repetitive based on searches using the *X*. *laevis* genome sequence version 10.1. There are two exons in *ccdc69-w* and we captured both.

**Fig 4:**
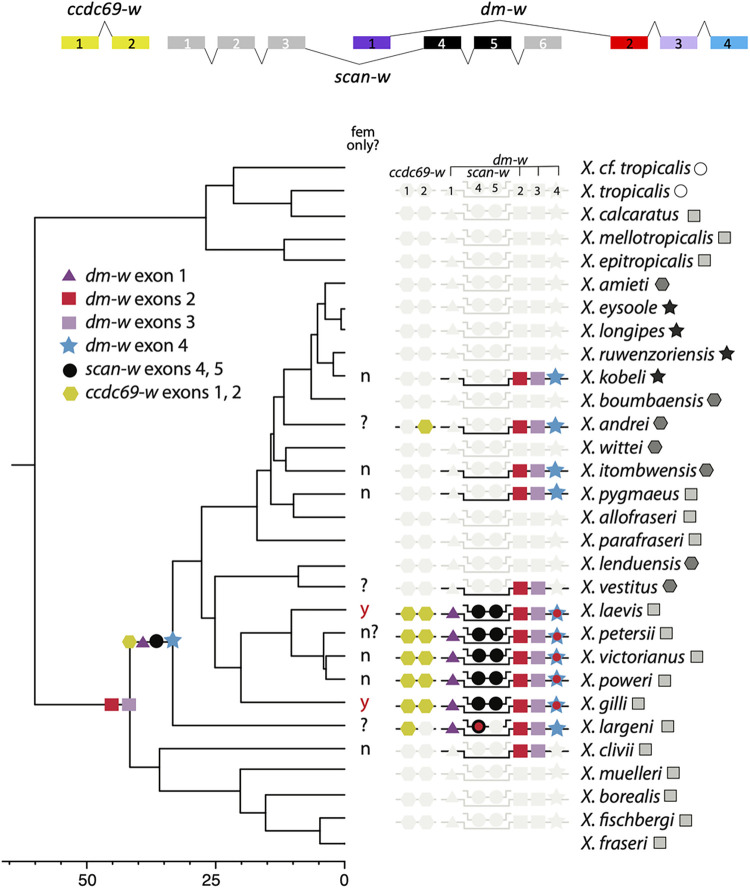
Targeted capture sequencing reveals evolutionary steps toward the female-determining genomic region of *X*. *laevis*. The genomic orientations of transcribed exons is depicted above a phylogenetic representation of the presence/absence data of capture data from exons 1 and 2 of *ccdc69-w*, exons 4 and 5 of *scan-w* and exons 1, 2, 3, and 4 of *dm-w*. Female specificity of *dm-w* (fem only?) is based on PCR assays [this study; 31] with question marks indicating species where female-specificity of *dm-w* is unknown, including for *X*. *petersii* where our PCR assay had inconsistent results. *Xenopus fraseri* and *X*. cf. *tropicalis* were not assayed by the capture sequencing. The order of numbered exons of each gene corresponds to their genomic locations, including overlapping transcribed regions of *scan-w* and *dm-w*; only captured exons are mapped on the phylogeny (limitations of “by-catch” data for *dm-w* exon 1 are discussed in main text). A red dot inside symbols indicates mutations that alter the reading frame as detailed in S1 Text. Data are plotted on a Bayesian phylogeny estimated from complete mitochondrial genomes [47] which does not reflect reticulating relationships among species that stem from allopolyploidation [38]. Ploidy level of each species is indicated by a circle (diploids), a square (tetraploids), a hexagon (octoploids), or a star (dodecaploids). Scale bar is in millions of years before the present, and almost all nodes have 100% posterior probability. See Evans et al. [47] for further details on phylogenetic estimation, node confidences, and confidence intervals of divergence estimates.

Capture sequencing of one individual (usually a female) from almost all *Xenopus* species identified *dm-w* exon 4 in *X*. *laevis*, *X*. *victorianus*, *X*. *poweri*, *X*. *petersii*, *X*. *gilli*, *X*. *pygmaeus*, *X*. *kobeli*, *X*. *itombwensis*, *X*. *andrei*, and *X*. *largeni*. The top BLAST hit of the *dm-w* exon 4 sequences that were capture sequenced matched the annotated exon 4 of this gene in the *X*. *laevis* version 10 genome sequence (S5 Table), which is consistent with our interpretation that these capture sequences were indeed *dm-w* exon 4. *Xenopus vestitus* and *X*. *clivii* are the only species in which *dm-w* exons 2 and 3 were previously detected [31] but where capture sequences reported in this study did not detect *dm-w* exon 4. These observations minimally indicate an origin of *dm-w* exon 4 prior to the diversification of the most recent common ancestor species that contain this exon (a blue star Fig 4). These results further suggest that *dm-w* exon 4 is not present in species that also lack *dm-w* exons 2 and 3 [31] and that *dm-w* exon 4 may have been lost in *X*. *vestitus* and possibly *X*. *clivii* (depending on when this exon became linked to *dm-w* exons 2 and 3; discussed further below). *Xenopus petersii*, *X*. *itombwensis*, and *X*. *andrei* had in-frame deletions in the coding region of *dm-w* exon 4, and *X*. *poweri* had a frameshift deletion near the end of the coding region of this exon (S1 Text); we did not attempt to assess the functional effects of these mutations.

Capture sequencing identified *scan-w* exons 4 and 5 in five species (*X*. *laevis*, *X*. *petersii*, *X*. *poweri*, *X*. *victorianus*, and *X*. *gilli*; Fig 4). We detected *scan-w* exon 4 but not exon 5 in *X*. *largeni*. Capture sequencing identified *ccdc69-w* exons 1 and 2 in seven species (*X*. *laevis*, *X*. *petersii*, *X*. *poweri*, *X*. *victorianus*, *X*. *gilli*, *X*. *largeni*, and *X*. *andrei*; Fig 4). BLAST results to the *X*. *laevis* genome were consistent with our annotations of these sequences (S5 Table). Capture sequencing of *scan-w* exon 4 also captured the sequences of *dm-w* exon 1 (which is non-coding) in each individual for which *scan-w* exon 4 was detected (*X*. *laevis*, *X*. *petersii*, *X*. *poweri*, *X*. *victorianus*, *X*. *gilli*, and *X*. *largeni*; S5 Table). This demonstrates that these exons of these genes are physically linked in these five species at least.

Capture sequencing additionally identified non-target sequences that are homologous to some of the targeted exons in various species (S5 Table). In *X*. *laevis*, for example, we identified exons 1 and 2 of *ccdc69*.*L* but not exons 1 and 2 of *ccdc69*.*S*, even though the genome assembly evidences both exons for both homeologs. This opens the possibility that the *X*. *laevis* sample used for capture sequencing lacked the *ccdc69*.*S* gene, though we cannot rule out the possibility that this is due to failure to capture this sequence (for example due to divergence of *ccdc69*.*S* from the capture probes).

*Scan-w* and *ccdc69-w* originated by gene duplication of autosomal loci [28], and we therefore interpret the detection of any portion of these genes as evidence that the entirety of these loci (i.e., all exons that are currently present in *X*. *laevis*) were present ancestrally. The capture data from *scan-w* and *ccdc69-w* thus indicate that all three of these genes became linked around the same time that *dm-w* exon 4, or even earlier if *scan-w* and *ccdc69-w* were either lost or undetected in *X*. *clivii* (Fig 4).

Some of the capture sequences had mutations that interrupted the reading frame (S1 Text). Overall, however, these capture results identify uninterrupted coding regions of exons 1 and 2 of *ccdc69-w* and exons 4 and 5 of *scan-w* in five species (*X*. *laevis*, *X*. *petersii*, *X*. *poweri*, *X*. *victorianus*, and *X*. *gilli*) and a subset of these exons and/or closely related paralogs in *X*. *largeni* and *X*. *andrei*.

### PCR assay for sex-specificity of *dm-w*

If *dm-w* is the trigger for female differentiation in *Xenopus* species in addition to *X*. *laevis*, then this gene is expected to be present in all females and no males. However, as discussed above, a previous PCR assay of six *Xenopus* species found *dm-w* to be female specific in *X*. *laevis* and *X*. *gilli* but not in *X*. *itombwensis*, *X*. *pygmaeus*, *X*. *clivii*, or *X*. *victorianus* [31]. We tested the female specificity of *dm-w* with a PCR assay in three additional species beyond those considered by [31]. These assays indicate that *dm-w* is not female-specific in *X*. *poweri* or *X*. *kobeli* and possibly not *X*. *petersii*, though the results in this last species were not conclusive due to inconsistent amplifications S6 Table. We also identified additional *X*. *victorianus* individuals beyond those previously identified [31] in which *dm-w* was not female-specific. With a handful of exceptions, for each individual independent attempts to amplify *dm-w* exons 2, 3, and 4 were generally all successful or all unsuccessful (S6 Table). This is consistent with these three exons being genetically linked and co-inherited. Based on these results and the consistent detection of all three exons in one female individual from several other species (Fig 4), we suspect these exons, when present, are genetically linked in other *Xenopus* species as well.

Results presented here and in [31]–which include capture sequencing of one individual (usually female) of almost all *Xenopus* species and PCR surveys of multiple male and female individuals of several *Xenopus* species–provide context into the evolution of female-specificity of *dm-w* in extant *Xenopus* species (Fig 4). These results suggest that female-specificity of *dm-w* is positively correlated with (i) the presence of exon 4, (ii) a derived extension of the coding region of *dm-w* exon 4 (due to mutation in an ancestral stop codon that extended the coding region; additional details are provided in S1 Text), and (iii) seemingly intact *scan-w* and *ccdc69-w* (for the exons examined here) on the ancestral genomic region that is female-specific in *X*. *laevis* (Fig 4). In *X*. *victorianus*, *X*. *poweri*, and possibly *X*. *petersii* the most parsimonious interpretation is that sex-specificity of *dm-w* was lost recently, presumably at some point after divergence from an ancestor of *X*. *laevis*.

## Discussion

We examined function and assembly of a W chromosome-specific genomic region in the African clawed frog *Xenopus laevis* that includes three genes (*dm-w*, *scan-w*, *ccdc69-w*). All three of these genes arose *de novo* by one or more independent small scale duplication events during diversification of *Xenopus* [this study; 28,35,36].

A striking finding to emerge from this study is that all genes in this W chromosome-specific genomic region either are or have been functionally dispensable. Rapid and pervasive degeneration of these genes is consistent with the expectation that the efficacy of natural selection is lower in non-recombining compared to recombining genomic regions [42,43]. In *X*. *laevis*, only *dm-w* is required to trigger female development and fertility, but not for viability, and *scan-w* and *ccdc69-w* are not essential for viability or female development and fertility. We note that this study does not demonstrate whether *dm-w* alone is sufficient to trigger female development because another (unidentified) factor could act upstream of *dm-w*. This possibility was tested using transgenic males that ectopically express *dm-w* [29] but, as discussed previously, sex reversal was observed only in a subset of transgenic males, possibly due to variable levels of transgene expression.

Comparisons across *Xenopus* species evidence dispensability of all three of these genes. Most descendant *Xenopus* species of the ancestor in which *scan-w* and *ccdc69-w* arose now carry truncated and perhaps non-functional versions of these genes, or appear to lack them altogether, and females that carry knockout mutations for *scan-w* or *ccdc69-w* are viable and fertile. Likewise, since its origin, several *Xenopus* species have lost *dm-w*, and several other species appear to retain it in a shorter (*X*. *clivii*, *X*. *vestitus*) and/or diminished form (compared to the ortholog in *X*. *laevis*) in which *dm-w* lacks a completely dominant female-determining function (*X*. *kobeli*, *X*. *itombwensis*, *X*. *pygmaeus*, *X*. *clivii*, *X*. *victorianus*, *X*. *petersii*, *X*. *poweri*) [this study; 31]. Thus, available information suggests that *dm-w* is the trigger for female differentiation in *X*. *laevis*, this gene became dispensable over relatively modest stints of evolution, with new mechanisms of sex determination abetting or replacing *dm-w* in several species. Below we discuss these findings in more detail, and their implications for understanding the origin and evolution of sex-specific genomic regions.

### Non-overlapping functional components of a W chromosome-specific genomic region

In principle, the origin of a sex-specific regions that contain multiple genes may be favored by natural selection if it binds together genetic variation with synergistic benefits. This is perhaps most obvious at the level of an individual gene that triggers sex determination, and where recombination suppression prevents intra-genic disruptions that could lead to neutered, intersex, or infertile offspring. Across multiple linked genes, synergy conceivably could be achieved through biological interactions (epistasis). That *dm-w*, *scan-w*, and *ccdc69-w* are all W chromosome-specific in *X*. *laevis* opens the possibility that a combination of some or all three of these loci are necessary for female differentiation, fertility, or viability. However, we recovered no evidence for strong epistatic effects among these three genes. Sex-specific genomic regions also have the potential to resolve sexual antagonism [12,15]; in this study we did not attempt to evaluate this possibility.

Our knockout lines demonstrate that only *dm-w* is required for female differentiation and fertility in *X*. *laevis* because genetic females with a non-functional *dm-w* gene develop into fertile sex-reversed phenotypic males. Genetic females that carry non-functional *scan-w* and *ccdc69-w* genes develop into fertile phenotypic females, which demonstrates that these two genes are not required for female differentiation, fertility, or viability. This extends previous work by demonstrating that full knockout of *dm-w* in *X*. *laevis* causes complete female to male sex reversal in all individuals and allows us to reject the notion that all three or any two of the W chromosome-specific loci in the *X*. *laevis* are essential for female differentiation or fertility. Our knockout lines thus support previous inferences based on the observation of partial sex reversal elicited by RNA interference of *dm-w* [29,30].

In fruit flies, 30% of newly evolved genes (which are typically also young) appear to be essential [48], which suggests that essential functions may arise quickly. Though *dm-w* is essential for female development and thus reproduction of *X*. *laevis*, *scan-w* and *ccdc69-w* are not. In several other *Xenopus* species, *dm-w* was replaced several times by novel but not yet known triggers for sex determination. These findings thus fail to provide support rapid evolution of essentiality in new genes.

Several insights into biological function of these W chromosome-specific genes can be gleaned from comparisons of the transcriptomes in the developing mesonephros+gonads at a crucial developmental junction (at tadpole stage 50) where *dm-w* is thought to initiate sexual differentiation [29]. At this early stage of sexual differentiation, relatively few genes were found to be significantly differentially expressed in the *dm-w* knockout line compared to wildtype sisters, and no significant enrichment of gene ontology was identified in differentially expressed genes in the *dm-w* knockout line (S1 and S2 Tables). This suggests that pronounced transcriptomic consequences of *dm-w* expression are realized later in development or that subtle (and undetected) changes in the transcriptome at this stage have mushrooming effects later during development. Consistent with this latter scenario, a focused analysis of differential expression of 74 sex-related genes demonstrates that the mesonephros+gonad transcriptome of the *dm-w* knockout is significantly masculinized at tadpole stage 50 (Figs 3 and S7–S8), even though most sex-related transcripts are not individually significantly differentially expressed.

Because they share a DNA binding domain and are co-expressed during development, *dm-w* is proposed to be a transcription factor that competitively binds to regulatory regions that are also recognized by the male-related gene *dmrt1* (from which *dm-w* is partially derived [29]), thereby inhibiting the initiation of male differentiation by *dmrt1* [30]. Antagonistic function analogous to that proposed for *dm-w* also exists in newly evolved partial paralogs of the *srgap2* gene that are involved in human cortical development [49,50] and in amphioxus where one paralogous estrogen receptor is activated by estrogen while another lost this ancestral function and acts as a repressor of the first [51]. An interesting direction for future work would be to evaluate how knockouts of *dmrt1*.*L* and *dmrt1*.*S* affect sexual differentiation and gene expression in *X*. *laevis* and the diploid species *X*. *tropicalis*, which could offer insights into whether subfunctionalization or neofunctionalization of these homeologs after allotetraploidization preceded the origin of *dm-w*.

In the mesonephros+gonad at tadpole stage 50, transcriptome masculinization was not observed in the *ccdc69-w* knockout line and there was only a weak signal masculinization in the *scan-w* knockout line. Gene ontology analysis of significantly differentially expressed genes in the *scan-w* and *ccdc69-w* lines suggest distinctive functions with unclear relevance to sexual differentiation (S3 Table). This suggests distinctive functional roles of these genes in comparison to *dm-w*. The functions of *scan-w* and *ccdc69-w* presumably overlap to some degree with those of their respective autosomal paralogs, but arguably are both substantially distinct from *dm-w* and from each other, and our findings suggest they minimally impact or are extraneous to female sexual differentiation. Taken together, these results point to distinctive biological functions of each of these W chromosome-specific genes, with effects of each gene that extend to diverse biological processes, cellular compartments, and developmental stages.

Only one gene–*capn5-z*–is found on the Z chromosome but not the W chromosome of *X*. *laevis* [28]. Wildtype females have one W and one Z chromosome and therefore have one *capn5-z* allele, whereas wildtype males have two Z chromosomes and two *capn5-z* alleles. This gene is expressed in both sexes in the developing gonads, and also in adult gonads, brain, and spleen, and to a lesser extent in several other tissues [heart, liver, stomach, mesonephros; 28]. That *dm-w* knockout individuals (W*Z individuals) develop into what appear to be phenotypically normal and fertile males, demonstrates that two alleles of *capn5-z* are not required for male development or viability in *X*. *laevis*. That W*W* knockout individuals also developed into phenotypic males suggests that *capn5-z* may not be required at all for male development; this possibility could be further explored with histology or fertility assays that we did not perform.

### Diverse origins and temporarily staggered assembly of a sex-specific genomic region

New genes arise from a variety of mechanisms, including horizontal gene transfer [52], gene duplication [53], exon shuffling [54], replication or modification by transposable elements [55], gene fusion [56] or fission [57], and *de novo* origin from previously non-coding genomic regions [58]. These diverse possible origins raise the question of how the three differently functioned genes on the W chromosome of *X*. *laevis* arose and become tethered together. As discussed above, the closest paralogs in the autosomes of *dm-w*, *scan-w*, and *ccdc69-w* are not tightly linked, which suggests that they have independent origins on the W chromosome-specific portion of the W chromosome [28,29,35–37]. Homeologs of exons 2 and 3 of *dm-w* (*dmrt1*.*L*, *dmrt1*.*S*) are on chr1L and chr1S at positions ~139 and 119 Mb in *X*. *laevis* genome assembly 10.1, respectively. Another part of the coding region of *dm-w* (in exon 4) arose independently from a non-coding transposon sequence, and homologous sequences of *dm-w* exon 4 are present on chromosomes 2L, 7L, and unplaced scaffolds [36]. Using Blast [59], we identified homeologs of *ccdc69-w* on chr3L (*ccdc69*.*L*) and chr3S (*ccdc69*.*S*) at positions ~21.5 and 7.6 Mb, respectively, and on chr5L (*LOC108716149*) at ~63.5 Mb on the *X*. *laevis* genome assembly version 10.1. Blast searches identified sequences with homology to *scan-w* in multiple genomic locations, including regions that are annotated as genes and regions that are not annotated. Despite its small size, this scattered genomic distribution of homology of these W chromosome-specific genes underscores remarkably diverse origins of this small genomic region of *X*. *laevis*.

Targeted capture sequencing reported here and elsewhere [31] demonstrates that the most recent common ancestor of species that carry *dm-w* exons 2 and 3 is older than the MRCA of species in which *dm-w* exon 4, *scan-w* exons 4 and 5, and *ccdc69-w* exons 1 and 2 were detected (Fig 4). We note that this inference depends on the phylogenetic placement of *X*. *clivii*; the placement of *X*. *clivii* depicted in the mitochondrial phylogeny presented in Fig 4 is consistent with that recovered from a phylogenetic analysis of over 1,000 expressed transcripts [60]. “By-catch” sequencing of the non-coding *dm-w* exon 1 with probes for *scan-w* exon 4 indicates that *dm-w* exon 1 was present in the most recent common ancestor of *X*. *laevis* and *X*. *largeni*, which is consistent with findings from another study [41]. Because we did not attempt to directly capture *dm-w* exon 1, these data do not allow us to determine whether this exon was also present in an even older ancestor. *Dm-w* exon 4 has an independent origin from exons 2 and 3 [36] and has previously been detected in *X*. *laevis*, *X*. *largeni*, *X*. *petersii*, *X*. *itombwensis*, and *X*. *pygmaeus* [29,31,36]. We extend these findings by identifying *dm-w* exon 4 in several more species (Fig 4), but notably we do not infer *dm-w* exon 4 to have been present in a more phylogenetically diverged species (such as *X*. *clivii* which carries *dm-w* exons 2 and 3 but not 4) as compared to previous inferences.

One interpretation of these data is that *dm-w* exons 2 and 3 appeared in the most recent common ancestor of *X*. *clivii* and *X*. *laevis*, and that *dm-w* exon 4, *scan-w*, *ccdc69-w*, and possibly *dm-w* exon 1 subsequently arose in the most recent common ancestor of *X*. *largeni* and *X*. *laevis*. Another interpretation is that all of these components were present in the most recent common ancestor of *X*. *clivii* and *X*. *laevis*, and that *dm-w* exon 4, *scan-w*, *ccdc69-w*, and perhaps *dm-w* exon 1 were later lost in *X*. *clivii*. This second scenario is less parsimonious than the first because it necessitates two deletions in an ancestor of *X*. *clivii* (one upstream of *dm-w* exons 2 and 3 to remove *scan-w*, and *ccdc69-w* and one downstream of *dm-w* exons 2 and 3 to remove *dm-w* exon 4). Either way, capture data suggests that subsequent evolution led to the loss of these genes–or portions of them–in various lineages (e.g., *ccdc69-w* exon 1 in *X*. *andrei*, *scan-w* exon 5 in *X*. *largeni*, *dm-w* exon 4 in *X*. *vestitus*).

A caveat to our interpretations of the targeted capture sequences is the possibility of false negatives, where a gene was not detected in some species even though it was present. This could happen if probe efficiency were low due to divergence between the probe and its target, or because an individual used in the library preparation happened to be sex reversed. However, the congruence between the results from different capture data for *dm-w* exons 2 and 3 [31], a PCR survey for these exons [35], and capture data from *dm-w* exon 4 (this study) is very high, with only two biologically plausible discrepancies (a failure to detect exon 4 in two species). For this reason, we suspect that the frequency of false negatives in our capture data is low. For species where our failure to detect *dm-w* accurately reflects an absence of this gene, sexual differentiation presumably is triggered by other unidentified factor(s).

With the exception of the “by catch” of *dm-w* exon 1 by our probes for *scan-w* exon 4, these capture sequences by themselves do not demonstrate that the captured sequences are physically linked on the same chromosome (apart from *X*. *laevis* where we know they are physically linked based on the genome assembly [28]). However, linkage of these exons in several other *Xenopus* species is supported by a PCR survey [31] that included 2–6 independent amplicons of different regions of *dm-w*, including portions of dm-w exons 2, 3, and 4, a non-transcribed region upstream of *dm-w*, and a portion of the coding region of *scan-w*. Although *dm-w* was not found to be female-specific in several species, independent attempts to amplify different portions of this gene in different samples from different species were generally all either successful or all unsuccessful [31], which is consistent with linkage, even in the absence of sex-specificity.

### Developmental systems drift

Developmental system drift refers to the origin of diverse genetic underpinnings for conserved traits across different species [61]. In sexual species, developmental pathways linked to sexual differentiation are crucial for reproduction but are orchestrated by diverse genes and genetic interactions, and are thus a prime example of developmental systems drift [61]. Findings discussed here and elsewhere [31] evidence developmental systems drift of sex-determination in *Xenopus* by demonstrating that *dm-w* is not female-specific in almost all species that carry this gene (Fig 4), even though it triggers female differentiation in *X*. *laevis* and possibly *X*. *gilli*. The phylogenetic distribution of female-specificity of *dm-w* suggests that the female determining capacity of *dm-w* was probably in place in the most recent common ancestor of *X*. *laevis* and *X*. *gilli*, but then lost by developmental systems drift in several closely related species such as *X*. *victorianus*. An alternative interpretation (that seems unlikely) is that the female determining capacity of *dm-w* arose independently (and convergently) in *X*. *laevis* and *X*. *gilli*.

One or more mutations extended the coding region of *dm-w* exon 4 of *X*. *laevis*, *X*. *gilli* and closely related species (S1 Text). Exon 4 increases the DNA-binding activity of *dm-w* in *X*. *laevis* [36] though it is not clear what the functional implications of the ancestral extension of the coding region may be. Even though the coding region of *dm-w* seems intact in *X*. *victorianus*, *X*. *poweri*, and *X*. *petersii* and includes the extended coding region in exon 4, female-specificity of *dm-w* was lost in some or all of these species based on our PCR surveys of several male and female individuals (results were inconclusive for *X*. *petersii*; S6 Table), thereby providing further evidence of developmental systems drift of genetic sex determination.

### Outlook

Key unanswered questions raised by these findings ask what the ancestral function of *dm-w* was when it arose, and whether and how *dm-w* influences sex determination in species where this gene is not female-specific (minimally *X*. *kobeli*, *X*. *itombwensis*, *X*. *pygmaeus*, *X*. *clivii*, *X*. *victorianus*, *X*. *poweri*, *X*. *petersii*). It remains unclear why *dm-w* appears to segregate as a single allele in *X*. *clivii*, *X*. *kobeli*, and several other species–which would explain why it is found in some female and male individuals but not others–as opposed to being a “regular” autosomal locus with two alleles in all individuals of both sexes, which is the case in *X*. *itombwensis* [31]. It is possible that *dm-w* was (and in some species is) an “influencer” of female differentiation in the sense that it tends to be found in females, but this also depends on variation at other loci. Because these downstream genes are autosomal, they also have been duplicated by allopolyploidization, which occurred several times independently in *Xenopus* to generate a diversity of tetraploids, octoploid, and dodecaploids species [46, 62, 63]. Due to differences in ploidy level, copy numbers of autosomal genes that interact with *dm-w–*such as *dmrt1 –*vary considerably; barring gene loss and pseudogenization, dodecaploid species such as *X*. *kobeli* carry six copies of autosomal genes (each with two alleles); octoploid species such as *X*. *itombwensis* carry four, and tetraploid species have two. Interestingly, pseudogenization of *dmrt1* homeologs has occurred independently multiple times in *Xenopus*, and in a phylogenetically biased fashion with more silencing of genes from one homeologous lineage (*dmrt1S*) than the other (*dmrt1L*) [35]. Clearly, further insights into these questions could be gained with experiments that explore function of homeologs of *dmrt1* and other duplicated sex-related genes in *X*. *laevis* and of *dm-w* in species where this locus is not female-specific.

## Methods

### Ethics statement

All work with live animals was approved by the Animal Use Committee at McMaster University (AUP# 17–12–43) and the Institutional Animal Care and Use Committee at the Marine Biological Laboratory (IACUC # 22–29).

### Knockout of *dm-w*, *scan-w*, and *ccdc69-w*

We generated knockout individuals using CRISPR/Cas9 [64]. Single guide RNAs (sgRNAs) were designed to target the beginning of the coding region for *dm-w*, *scan-w*, and *ccdc69-w* using CRISPRdirect (https://crispr.dbcls.jp/) with an aim of maximizing disruption of protein function (S7 Table). The specificity of our guides was evaluated using the *X*. *laevis* genome assembly 9.1. Single stranded guide RNA (sgRNA) was generated from a DNA template that contained a promoter (SP6 for *dm-w* and T7 for *scan-w* and *ccdc69-w*) and a universal reverse primer for subsequent transcription. The DNA template was then used for sgRNA production using the Megascript SP6 or T7 kit (Invitrogen, Thermo Fisher Scientific).

SgRNAs were injected with the Cas9 protein into one cell embryos from *X*. *laevis* J-strain individuals. Because cutting generally happens after several rounds of cell division, the resulting F0 embryos are mosaics of wild-type and mutant cells. F0 phenotypic females (in the case of *scan-w* and *ccdc69-w*) or phenotypic males (in the case of *dm-w*) were then back-crossed to wildtype (J strain) males or females respectively. Mutations were confirmed by sequencing and the genetic sex was verified by amplification of other W-specific genes and by surgical inspection of gonads after euthanasia. F1 individuals were also crossed to wild-type individuals to evaluate fertility, with ovulation (phenotypic females) or clasping (phenotypic males) facilitated by injection of human chorionic gonadotropin (Sigma).

For all three genes, sequence chromatograms of F0 individuals had overlapping sequences that begin at the targeted region and that disrupted the putative open reading frame of each gene. Because cutting occurs at a multicell stage of embryogenesis, overlapping sequences were expected due to a mosaic genotype comprising wild-type and mutant sequences. These F0 females were then crossed with wild-type (J-strain) males to generate non-mosaic F1 knockout individuals, which were confirmed by Sanger sequencing (S1 Fig).

### Transcriptome analysis of F1 progeny

With an aim of better understanding the functions of *dm-w*, *scan-w*, and *ccdc69-w*, we compared transcriptomes of the developing mesonephros+gonad of knockout individuals to developmental-stage-matched wildtype sisters that were co-reared in the same tank. We focused on tadpole stage 50, which is when gonadal differentiation is thought to be initiated because the gonads are not differentiated at this stage and because an increase in expression of *dm-w* at this stage precedes gonadal differentiation thereafter [29]. Tadpole stage 50 was determined based on morphological attributes including the shape of the head, size of tentacles, and size and shape of rear limb buds [65,66]. The genotypic sex of the tadpoles was assessed by amplifying the three known W chromosome-specific genes (*dm-w*, *scan-w*, and *ccdc69-w*) with successful amplifications in all three genes used to identify genetic females. Mutant and wildtype individuals were then distinguished by sequencing the mutant gene for each line.

We compared transcriptomes from each knockout line to stage-matched wildtype sisters that were co-reared in the same tank. For the *dm-w*, *scan-w* and *ccdc69-w* knockout lines, mesonephros+gonadal transcriptomes from six, five, and six knockout individuals, and six, four, and two wildtype females were analyzed. To further understand the transcriptomic consequences of our gene knockouts, we established a baseline expectation for sex-biased gene expression using three independent batches of wildtype male and female mesonephros+gonad transcriptomes that were derived from three independent clutches of siblings at tadpole stage 50. The MF1, MF2, and MF3 batches included two, three, or six females and six, five, or six males, respectively. The wildtype females in the MF1 of the sex-biased expression analysis were the same as those in the *ccdc69-w* knockout versus wildtype analysis; data from the MF2 and MF3 batches were from different clutches from each other and from all other analyses. For the *dmw* dataset, four wildtype females were run on a different lane from the other samples. For the *ccdc69-w* and MF2 datasets, three wildtype males from each dataset were run on a different lane from the other samples. For the MF3 dataset, three wildtype females and three wildtype males were run on a different lane from the other samples. Because of this sampling distribution, we were only able to control for possible lane effects in the design of the MF3 analysis.

RNA quality was assessed for each sample using an Agilent Bioanalyzer; we selected samples with an RNA integrity number [67] of at least 8.5 out of 10 for analysis (median = 9.6). RNAseq libraries were generated using Clontech/Takara SMARTer v4 cDNA conversion kit followed by the Illumina Nextera XT library preparation. Paired-end sequencing (150 bp) was performed on portions of three lanes of an Illumina Novaseq 6000 machine. Adapters and reads of poor quality and short length were removed using Trimmomatic v. 0.39 [68] with settings that retained reads of at least 36 bp and with an average quality per base higher than 15 on a sliding window of 4 bp; bases of poor quality (below 3) at the start and end of a read were also removed. After trimming this resulted in an average of 46.9 million (*dm-w*), 45.6 million (*scan-w*), and 54.6 million (*ccdc69-w*) paired-end reads per sample. These data have been deposited in the NCBI SRA (BioProject PRJNA989530).

For each analysis of differential expression, we quantified transcript abundance in the *X*. *laevis* transcriptome reference version 10.1 using a mapping method: STAR version 2.7.9a [69], and a pseudocount method: Kallisto version 0.46.1 [70]. Counts from each method were processed with EdgeR version 3.16 [71] and DeSeq2 version 1.34.0 [72] to perform the analysis of differential expression. Prior to analysis of differential expression, genes with an average of less than two reads per individual were removed. Transcripts and genes were considered differentially expressed if the false detection rate adjusted p-value was less than 0.10.

We then performed a gene ontology analysis on each set of differentially expressed genes. Unfortunately, the annotations for the latest version of the *X*. *laevis* transcriptome are incomplete with many of the differentially expressed genes lacking a functional annotation and instead having unknown annotations that begin with “LOC” (S2 Table). Thus, for each quantification method and analysis of differential expression, we extracted the sequence of each differentially expressed gene and used the discontiguous blast algorithm [59] to identify putative orthologs (based on the best bit score) in a human transcriptome GRCh38.p13 release 42 [73]. This approach increased the number of annotated transcripts and the annotations of putative human orthologs generally matched the available annotations of *X*. *laevis* transcripts (S2 Table). We then used the gene ontology resource (http://geneontology.org/) to perform gene ontology analyses of biological function, molecular function, and cellular component, with significant enrichment based on Fisher’s exact test with a false discovery rate of 0.05.

### Sex related genes and transcriptome masculinization

To further evaluate whether and to what degree each knockout line (each of which are genetically female) has signatures of transcriptome masculinization, we examined correlations between the log2 transformed expression ratios of 74 sex-related genes [S3 Table; 44] between each pairwise comparison between six analyses of differential expression (i.e., three comparisons between wildtype male and wildtype female transcriptomes and three comparisons between knockout female and wildtype female transcriptomes). The expression data for these 74 sex related genes was obtained from the transcriptomic/RNAseq data. These correlations were calculated for each of the four RNAseq analysis pipelines that we performed (Kallisto + EdgeR, Salmon + EdgeR, Salmon + DeSeq2, and Kallisto + DeSeq2). For this analysis, no filtering was performed based on transcript abundance; instead we excluded outliers, defined as 1.5 times the interquartile range above or below the upper or lower quartile. Spearman’s correlation was calculated between the non-outlier log2 fold changes for each pairwise comparison and a *p-*value for this coefficient was calculated using the cor() function in R, which assumes the samples follow independent normal distributions.

If a knockout mutation (*dm-w*, *scan-w*, or *ccdc69-w*) led to masculinization of the mesonephros+gonad transcriptome, we expected a higher correlation between the log2 fold changes from the knockout analyses and one or more of the analyses of sex-biased expression in the wildtype transcriptomes. To test this, 1000 permutations were performed where the correlation between the non-outlier log2 fold changes of 74 randomly selected genes was calculated and compared to the observed. A *p-*value was calculated as 1 minus the rank of the observed correlation in the permutated correlations, divided by 1001.

### Phenotyping of knockout progeny

The phenotype of each knockout line was ascertained with respect to (1) phenotypic sex, (2) fertility, and (3) testis histology (if present). Phenotypic sex was assessed either surgically by inspecting gonads after euthanasia or based on ability to lay eggs after injection with 400 international units of human chorionic gonadotropin. Fertility was assessed by crossing mutant individuals with wildtype individuals of the opposite phenotypic sex and examining whether embryos were produced. Crosses were achieved by injection of 400 or 300 international units of human chorionic gonadotropin in phenotypic female or male individuals, respectively. Testis histology was examined using 4 μm sections of formalin-fixed paraffin-embedded tissues that were stained with a Leica Autostainer XL using Hematoxylin 560MX and Eosin 515LT SelecTech stains (Leica).

### Targeted next-generation sequencing and Sanger sequencing of W-specific and autosomal loci

We used targeted next-generation sequencing to assess presence, absence, and sequence variation of *dm-w* exon 4, *scan-w* exons 4 and 5, and both exons of *ccdc69-w* in 28 of 29 *Xenopus* species using the same panel of individuals and genomic DNA libraries as detailed previously [31]. To enrich the genomic libraries, we used 82 bp probes that overlap with 2 bp tiling (GenScript) that were designed based on exons of interest in *X*. *laevis*. Universal flanking sequences were added to each probe [74] and the probes synthesized on a 12k oligonucleotide array (GenScript). The oligonucleotide pool was then amplified by PCR and converted into single-stranded biotinylated DNA probes for in-solution hybridization capture using the method of [74]. The libraries were multiplexed, and paired end sequencing was performed on a portion of one lane of an Illumina HiSeq 2500 machine, with 125 bp paired-end reads. Sequences from each species were demultiplexed, assembled using Trinity 2.5.1 [75], and captured exons were identified using blastn [59]. Due to repetitive regions in *scan-w*, a 300 bp cutoff on all blast hits was applied. Sequences from each exon were aligned using MAFFT version 7.271 [76], adjusted manually, and manually inspected for putatively chimerical sequences. Our alignment included reference sequences from the *X*. *tropicalis* genome assembly 10.1 and *X*. *laevis* genome assembly 9.2 for each exon plus 200 bp upstream and downstream. Assembled capture sequences are deposited in GenBank (accession numbers are in S5 Table).

PCR assay and Sanger sequencing were also performed to evaluate the female-specificity of *dm-w* in three additional species beyond those evaluated previously [31]: *X*. *kobeli*, *X*. *petersii*, and *X*. *poweri* and additional *X*. *victorianus* individuals from two geographical areas. Amplification of a portion of the mitochondrial 16S ribosomal RNA gene was used as a positive control for each DNA extraction using primers 16Sc-L and 16Sd-H [77] and negative (no DNA) controls were performed for all amplifications. The phenotypic sex of each specimen of each species was determined surgically by inspecting gonads after euthanasia. For each individual, independent amplifications of *dm-w* exons 2, 3, and 4 were attempted and in individuals with unexpected amplifications (positive amplifications in males, negative amplifications in females) multiple independent amplifications were attempted.

## Supporting information

S1 Text Supplementary background and results. (DOCX)

S1 Fig Inactivation of the W-specific genes (a) *dm-w*, (b) *scan-w*, and (c) *ccdc69-w*. Gray boxes represent exons of each gene, black lines between these boxes are 5’ and 3’ untranslated regions and introns, and the positions of start and stop codons are indicated with an arrow and the word “stop” respectively. Sequences are shown for wildtype (wt), mosaic F0 individuals (F0), and knockout individuals (F1). Black bars underscore deletions and start codons are highlighted in pink for (a) and (c). These mutations are all within the coding region and result in a premature stop codon.
(TIF)

S2 Fig Testis histology of wildtype males (a-d) and sex reversed F1 females (e-h) carrying a *dm-w* knockout mutation. Black bars are 50 μm; individuals identification numbers are (a) 17E6, (b) 17F0 (c) 184B, (d) 1815, € 180A, (f) 180B, (g) 1844, (h) 1847. In (a) and (h) dotted circles indicate the margins of seminiferous tubules and arrows indicate clusters of late spermatids.
(TIF)

S3 Fig Expression of *dm-w* in females (f) and males (m) from each wildtype batch (MF1, MF2, MF3) and wildtype (wt) and knockout (ko) females from each experimental batch (dmw, scanw, ccdc). Count data from the two wildtype females in the MF1 batch are the same as in the ccdc batch. These data are from counts from STAR that were normalized with EdgeR; a normalized count of zero corresponds to less than -26.
(TIF)

S4 Fig Venn diagrams showing the numbers of overlapping and batch-specific differentially expressed genes with quantification using STAR and analysis of differential expression using DeSeq2. Labeling corresponds with Fig 2.
(TIF)

S5 Fig Venn diagrams showing the numbers of overlapping and batch-specific differentially expressed genes with quantification using Kallisto and analysis of differential expression using DeSeq2. Labeling corresponds with Fig 2.
(TIF)

S6 Fig Venn diagrams showing the numbers of overlapping and batch-specific differentially expressed genes with quantification using Kallisto and analysis of differential expression using edgeR. Labeling corresponds with Fig 2.
(TIF)

S7 Fig Analysis of transcriptome masculinization using the Kallisto-DeSeq2 pipeline. Labeling follows Fig 3.
(TIF)

S8 Fig Analysis of transcriptome masculinization using the STAR-DeSeq2 pipeline. Labeling follows Fig 3.
(TIF)

S9 Fig Analysis of transcriptome masculinization using the Kallisto-EdgeR pipeline. Labeling follows Fig 3.
(TIF)

S1 Table Significantly differentially expressed transcripts in the mesonephros/gonad of each of three comparisons between wildtype males and females (MF1, MF2, MF3) and each of three comparisons between a knockout line and wildtype siblings (*dm-w*, *scan-w*, *ccdc69-w*). Analysis of differential expression were performed for two quantification method (Kallisto, STAR) and two analysis method (EdgeR, DeSeq2) for a total of four pipelines, the results of which are all listed. The log2 fold change (logFC) and false detection rate P-value is indicated for each significantly differentially expressed gene (FDR). For all comparisons, wildtype female expression is the reference and thus the denominator of the log2FC. For some analyses, significant FDR corrected P-values are listed as NA because the mean normalized counts were lower than the default threshold. When identified, the gene acronym of the putative human ortholog is listed (Human).
(XLSX)

S2 Table Gene ontology analysis of differentially expression genes in the developing gonads for three knockout lines (*dm-w*, *scan-w*, *ccdc69-w*) compared to wildtype sisters, and for wildtype males compared to wildtype females (MF1, MF2, MF3). Results are listed for three gene ontology categories (biological process, molecular function, cellular component); subcategories with significant enrichment follow their parent category and are indicated wi“h”">"s, which reflect the degree of nestedness. For each gene analysisyis, the number of differentially expressed genes is indicated (# DE) and NS indicates no significant enrichment. Analyses were performed for each quantification method (Kallisto, STAR) and each analysis method (EdgeR, DeSeq2) and the false detection rate P-value is indicated for each significantly enriched annotation (FDR). Because a putative human ortholog was not identified for some transcripts (S1 Table), the number of genes used in the gene ontology analysis was generally lower than the number of differentially expressed genes.
(XLSX)

S3 Table Sex-related genes used to evaluate whether knockout mutations led to masculinization of gonad transcriptomes in tadpole stage 50. All genes were obtained from Piprek et al. (2018) [1] with the exception of the estrogen receptor. *Dm-w* was deliberately not included in this list because this gene was knocked out in one of our mutant lines.
(XLSX)

S4 Table Description of the samples used for the capture sequencing (Species, Field and Museum ID, Locality, Ploidy, Sex) and the number of reads (Reads) obtained from each library. Reads include sequences captured by probes described here (*dm-w* exon 4, *scan-w* exon 4 and 5, *ccdc69-w* exons 1 and 2) and also probes for other regions (*scan-w* exons 1–3, 6, androgen receptor exons 1–8, and *SRY*-related HMG-box exon 1) that were not included in this study.
(XLSX)

S5 Table Summary of capture sequences including locus, best blast hit of a representative query to *X*. *laevis* version 10 genome sequence or the *X*. *tropicalis* version 10 genome sequence where indicated (XT), species that carry this locus, and GenBank accession numbers. The query sequence was from the first species listed; some queries had multiple similarly optimal matches (multiple). Some species names are followed by additional details in parentheses.
(XLSX)

S6 Table Amplifications of dm-w exons 2, 3, and 4 demonstrate female specificity in our samples of X. petersii but not X. victorianus or X. kobeli. For each sample, mitochondrial DNA was amplified as a positive control (mtDNA); amplifications were either successful (Y), unsuccessful (N), or faint (Faint). Notes highlight consistent deviations from female-specificity.
(XLSX)

S7 Table Guides and primers used in this study; all listed in 5’ to 3’ orientation. Guide sequences include T7 or SP6 promoter sequences as indicated.
(XLSX)

S1 Data Data for Fig 2. (XLSX)

S2 Data Data for Fig 3. (XLSX)

S3 Data Data for S3 Fig. (XLSX)

S4 Data Data for S4 Fig. (XLSX)

S5 Data Data for S5 Fig. (XLSX)

S6 Data Data for S6 Fig. (XLSX)

S7 Data Data for S7 Fig. (XLSX)

S8 Data Data for S8 Fig. (XLSX)

S9 Data Data for S9 Fig. (XLSX)
