# Supplementary material for: Functional dissection and assembly of a small, newly evolved, W chromosome-specific genomic region of the African clawed frog Xenopus laevis
Source: PLoS Genet. 2023 Oct 4;19(10):e1010990. doi: 10.1371/journal.pgen.1010990 (PMC10578606; doi:10.1371/journal.pgen.1010990)
Supplement: S1 Text — (DOCX) [file pgen.1010990.s001.docx]

**Supplementary Background**

**Function and genomic structure of the W chromosome-specific region of *X. laevis***

Little is known about the other two female-specific genes on the W chromosome (*scan-w*, *ccdc69-w*) apart from their genomic locations and patterns of expression [1]. Similar to *dm-w*, both are expressed in female tadpole gonads during gonadal differentiation and also in adult ovary [1], which opens the possibility that they are involved with ovarian differentiation or function. *Scan-w* is expressed in adult brain and stomach, and *ccdc69-w* is expressed in adult brain [1]. These W-linked genes originated by independent gene duplication events of autosomal loci – *scan.L* or *scan-like* and *ccdc69.L*, respectively [1]. They are predicted to encode for proteins containing a scan and coiled-coil domains respectively. In *X. laevis* the autosomal copies of *ccdc69-w*, *ccdc69.L* and *ccdc69.S*, encode for proteins potentially involved in the assembly of spindle midzone, while various *scan*-like proteins are involved in cell differentiation and survival.

*Dm-w* has 4 exons; its coding region begins in exon 2 and ends in exon 4 [2]. This gene has a chimerical origin. Exons 2 and 3 are derived from the male-related homeolog *dmrt1-S* [3], which is an important transcription factor with male-related function in many metazoans [4], whereas exon 4 originated from a noncoding transposon, and exon 1 also appears to have an independent and as yet uncharacterized origin from the other exons [5].

*Dm-w* exons 2 and 3 are also present in other *Xenopus* species but are not always female-specific [3, 6]. In some *Xenopus* species, *dm-w* is also found in some males, and in at least one species (*X. itombwensis*), *dm-w* is present in all individuals and appears to have autosomal segregation [6]. The variation in female-specificity suggests dynamic functional evolution of *dm-w*, that this gene is female-specific in only a subset of *Xenopus* species that carry this gene, and that female specificity and/or the entire *dm-w* gene has been lost during *Xenopus* diversification [6]. Sequences of *dm-w* exons 2 and 3 are monophyletic with respect to *dmrt1L* and *dmrt1S* [3], which is consistent with a single origin for these portions of this gene.

*Dm-w* and *dmrt1L* and *dmrt1S* may competitively interact to influence expression of downstream genes [7]. Interestingly, however, *dmrt1L* and *dmrt1S* are autosomal and thus duplicated by polyploidization events. Without gene loss, tetraploid, octoploid, and dodecaploid species are expected to have two, four, or six *dmrt1* genes (each with two alleles). Interestingly, however, *dmrt1* homeologs have been lost in several species and *dmrt1S* homeologs were independently lost more frequently than *dmrt1L* homeologs [3]. That the dosages of *dm-w* and *dmrt1* alleles varies extensively among species could be a factor in the loss or non-female-specificity of *dm-w* in some species [this study; 6].

**Supplementary Methods**

**Knockout mutations**

We used CRISPR/Cas9 to introduce mutations that disabled function in *dm-w*, *scan-w*, and *ccdc69-w* (Fig. S1). For *dm-w*, F1 individuals had a 10 bp deletion starting at nucleotide position 31 which created a frameshift mutation in the predicted coding region (195 codons, not including the stop codon) and an early stop codon at codon number 37. As a result of this mutation, only the first nine amino acids are predicted to match the wildtype *dm-w*. For *scan-w*, F1 individuals had a 20 bp deletion beginning at nucleotide position 26 in the predicted coding region (628 codons), which created an early stop codon after codon number 31. As a result of this mutation, only the first nine amino acids are predicted to match the wildtype *scan-w*. For *ccdc69-w*, three types of F1 individuals were produced that carried either a 6 or 22 bp deletion in the predicted coding region (48 codons) or a combination of a 214 bp deletion and 12 bp insertion in the predicted coding region. The 6 bp deletion is an in-frame mutation and was not analyzed. The 12bp, 22 bp, and the 214 bp deletions all removed the predicted start codon.

**Supplementary Results**

**Transcriptomic analysis**

As a sanity check, we examined levels of *dm-w* expression in females and males in each of the wildtype batches and the wildtype and knockout individuals from each of the experimental batches (Fig. S3). As expected, males had zero or almost zero expression, with non-zero values presumably due to mis-mapping. In females, expression of *dm-w* was lower in MF1 than in MF2 and MF3. Variation in *dm-w* expression among wildtype and experimental females – including some females where little or no expression was detected – points to contributions of technical and/or biological variation in these expression data.

**Coding regions of genes in the W chromosome-specific region of *X. laevis***

Interpretation of nucleotide sequences of *dm-w* exon 4 in a phylogenetic context indicates that a stop codon was present in the most recent common ancestor of all species that have this exon that terminated translation after 42 codons in this exon. However, in the most recent common ancestor of *X. laevis* and *X. gilli*, a mutation changed this stop codon to a codon for glycine (*X. laevis*, *X. victorianus*, *X. petersii*, *X. poweri*) or glutamine (*X. gilli*), thereby extending the coding region by 28 amino acids. The presumptive ancestral stop codon is still present in several species (*X. largeni*, *X. pygmaeus*, *X. kobeli*, *X. andrei*, *X. itombwensis*).

In the *X. petersii* female that was used for capture sequencing, there was a 30 bp in-frame deletion in the beginning of *dm-w* exon 4, although the stop codon is homologous to that in other closely related species (*X. laevis*, *X. victorianus*, *X. poweri*, *X. gilli*). This deletion in *X. petersii* removes 10 amino acids in the translated protein. *Xenopus itombwensis* and *X. andrei* shared a 36 bp in-frame deletion near the stop codon. In the *X. poweri* female that was used for capture sequencing, a 16 bp frameshift deletion near the end of the exon introduces another novel stop codon that causes a loss of four amino acids at the C-terminus of translated protein relative to other closely related species such as *X. laevis*, and the incorporation of six different amino acids at the end of the peptide.

*Scan-w* exon 4 of *X. largeni* had a 1 bp frameshift mutation within the coding region and, as detailed above, *scan-w* exon 5 was not identified in *X. largeni*. This suggests that this gene is (minimally) truncated in *X. largeni*. In the other species where this gene was detected, the coding regions of exons 4 and 5 are uninterrupted. *Scan-w* exon 5 had a 12 bp in-frame deletion in *X. petersii*. Interestingly, 310 bp of *scan-w* exon 3 was previously (and unknowingly) sequenced in *X. gilli*, *X. laevis*, *X. poweri*, and *X. victorianus* (but not *X. petersii*) before the annotation of *scan-w* was available [8]. All of 24 *X. victorianus* individuals that were sequenced had a one bp deletion, which creates a frameshift mutation and an early stop codon in *scan-w*. In addition, twelve *X. victorianus* individuals carried a -10 bp deletion downstream of the one bp deletion. This study suggests that *scan-w* is not required for feminization – at least in *X. laevis* – but it is interesting that loss of female-specificity of *dm-w* was evolutionarily coupled with degeneration of another female-specific gene.

*Ccdc69-w* exon 1 is homologous to exon 8 of *ccdc69.L* and *ccdc69.S*; *ccdc69-w* exon 2 is homologous to exon 9 of *ccdc69.L* and *ccdc69.S*. Apart from the 173 bp insertion in the *X. laevis* sequence of *ccdc69-w* exon 1, none of the captured sequences for (i) *ccdc69-w* exon 1, (ii) the homologous regions of exon 8 of *ccdc69.L* and *ccdc69.S*, (iii) *ccdc69-w* exon 2, or (iv) the homologous regions of exon 9 of *ccdc69.L* and *ccdc69.S* had mutations that would interrupt the reading frame. The 173 bp insertion is flanked by intronic recognition sequences (5’ GT and 3’ AG) and may be an intron.

**Homologous capture sequences**

In addition to the capture results detailed in the main text, we identified other sequences that are presumably paralogous to *dm-w* exon 4, *scan-w* exons 4 and 5, and *ccdc69-w* exons 1 and 2 (Table S4). For homologs of *dm-w* exon 4, paralogous sequences were identified in several species that had *dm-w* exon 4 (*X. laevis*, *X. poweri*, *X. petersii*, *X. kobeli*, *X. vestitus*, *X. gilli*) and in some species that lacked this exon (*X. borealis*, *X. muelleri*, and *X. clivii*).

No paralogs of *scan-w* exon 4 were recovered by capture sequencing but we did identify a partial paralog of the 3’ half of *scan-w* exon 5 in *X. itombwensis* (Table S4). For exons 1 and 2 of *ccdc69-w*, capture sequencing also identified exon 1 of *ccdc69* from subgenus *Silurana* and paralogs of *ccdc69.L* and *ccdc69.S* in multiple *Xenopus* species (Table S4).

**References**

1. Mawaribuchi, S., et al., *Sex chromosome differentiation and the W- and Z-specific loci in Xenopus laevis.* Developmental Biology, 2017. **426**: p. 393–400.

2. Yoshimoto, S., et al., *A W-linked DM-domain gene, DM-W, participates in primary ovary development in Xenopus laevis.* Proceedings of the National Academy of Sciences, 2008. **105**(7): p. 2469–2474.

3. Bewick, A.J., D.W. Anderson, and B.J. Evans, *Evolution of the closely related, sex-related genes DM-W and DMRT1 in African clawed frogs (Xenopus).* Evolution, 2011. **65**(3): p. 698–712.

4. Zarkower, D., *Establishing sexual dimorphism: Conservation amidst diversity?* Nature Reviews Genetics, 2001. **2**(3): p. 175-185.

5. Hayashi, S., et al., *Neofunctionalization of a noncoding portion of a DNA transposon in the coding region of the chimerical sex-determining gene dm-W in Xenopus frogs.* Molecular Biology and Evolution, 2022. **39**(7): p. msac138.

6. Cauret, C.M., et al., *Developmental systems drift and the drivers of sex chromosome evolution.* Molecular Biology and Evolution, 2020. **37**: p. 799–810.

7. Yoshimoto, S., et al., *Opposite roles of DMRT1 and its W-linked paralog, DM-W, in sexual dimorphism of Xenopus laevis: implications of a ZZ/ZW-type sex-determining system.* Development, 2010. **137**: p. 2519–2526.

8. Furman, B.L., et al., *Pan-African phylogeography of a model organism, the African clawed frog 'Xenopus laevis'.* Mol Ecol, 2015. **24**(4): p. 909-25.
