# Supplementary figures and images for: Functional dissection and assembly of a small, newly evolved, W chromosome-specific genomic region of the African clawed frog Xenopus laevis
Source: PLoS Genet. 2023 Oct 4;19(10):e1010990. doi: 10.1371/journal.pgen.1010990 (PMC10578606; doi:10.1371/journal.pgen.1010990)

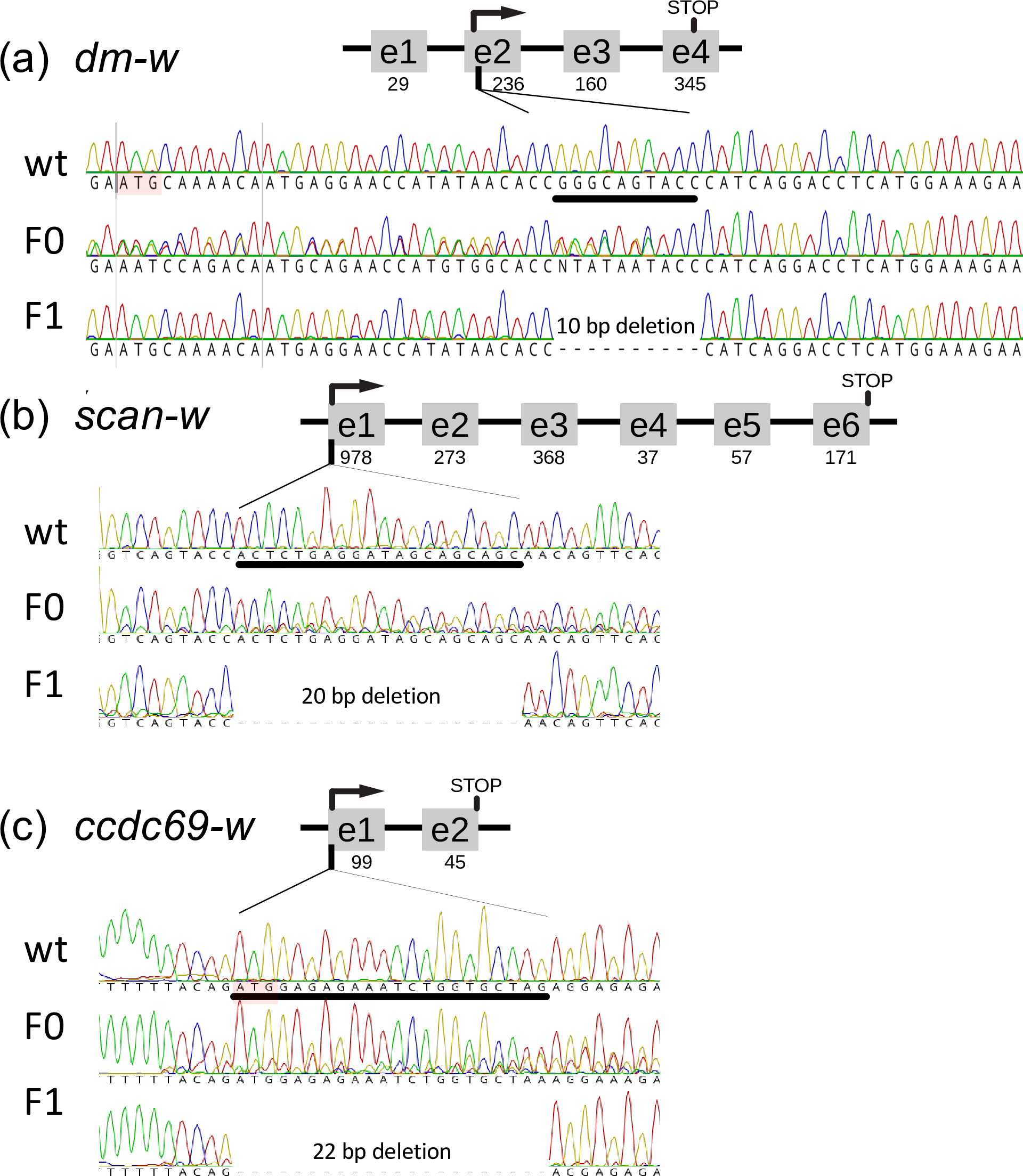

Supplement: S1 Fig — Inactivation of the W-specific genes (a) dm-w, (b) scan-w, and (c) ccdc69-w. Gray boxes represent exons of each gene, black lines between these boxes are 5’ and 3’ untranslated regions and introns, and the positions of start and stop codons are indicated with an arrow and the word “stop” respectively. Sequences are shown for wildtype (wt), mosaic F0 individuals (F0), and knockout individuals (F1). Black bars underscore deletions and start codons are highlighted in pink for (a) and (c). These mutations are all within the coding region and result in a premature stop codon. (TIF) [file pgen.1010990.s002.tif]

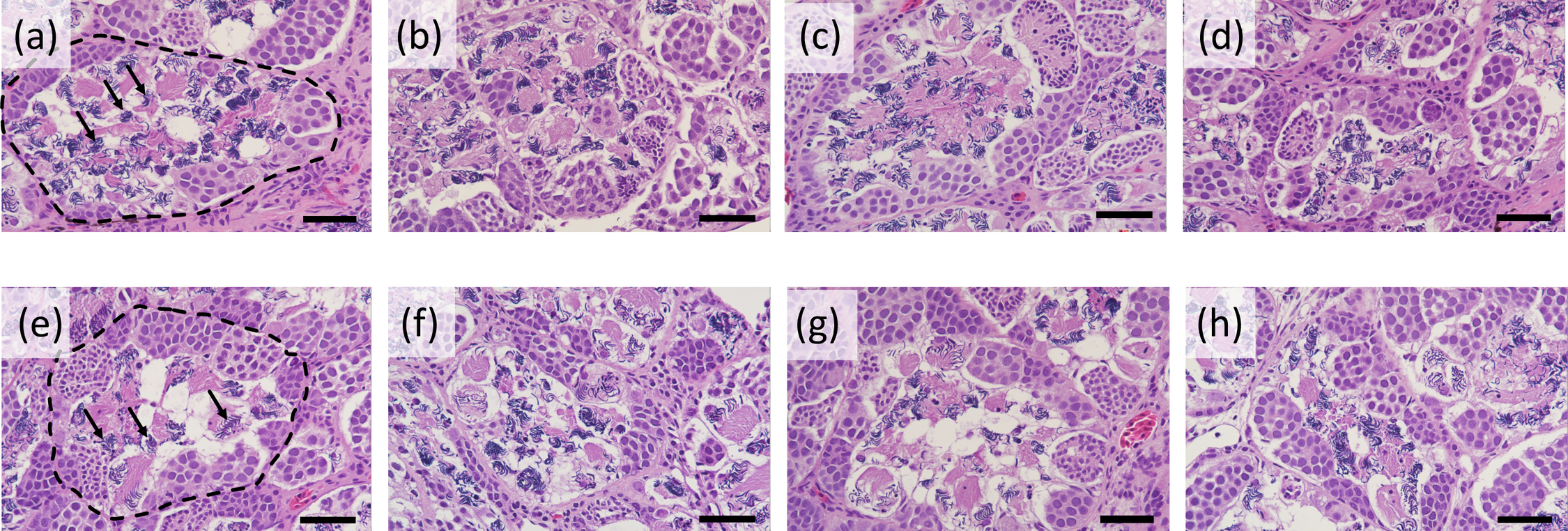

Supplement: S2 Fig — Testis histology of wildtype males (a-d) and sex reversed F1 females (e-h) carrying a dm-w knockout mutation. Black bars are 50 μm; individuals identification numbers are (a) 17E6, (b) 17F0 (c) 184B, (d) 1815, € 180A, (f) 180B, (g) 1844, (h) 1847. In (a) and (h) dotted circles indicate the margins of seminiferous tubules and arrows indicate clusters of late spermatids. (TIF) [file pgen.1010990.s003.tif]

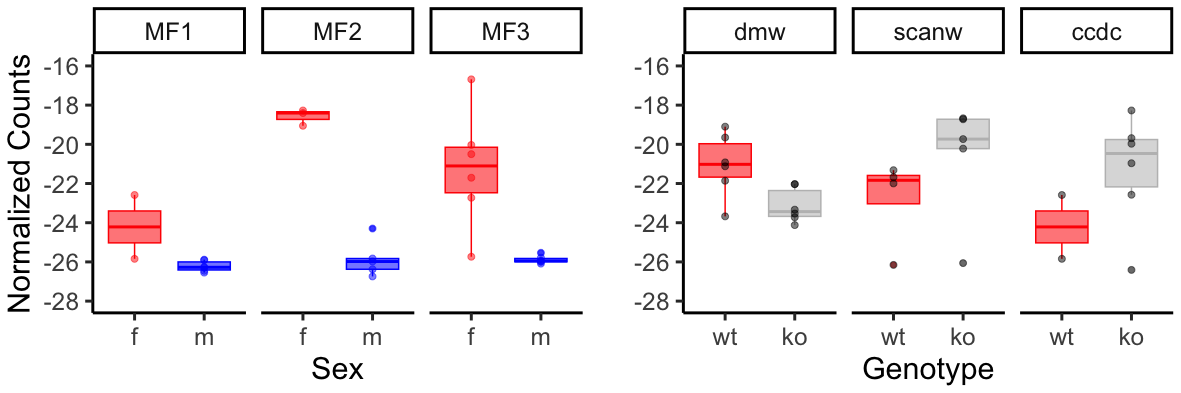

Supplement: S3 Fig — Expression of dm-w in females (f) and males (m) from each wildtype batch (MF1, MF2, MF3) and wildtype (wt) and knockout (ko) females from each experimental batch (dmw, scanw, ccdc). Count data from the two wildtype females in the MF1 batch are the same as in the ccdc batch. These data are from counts from STAR that were normalized with EdgeR; a normalized count of zero corresponds to less than -26. (TIF) [file pgen.1010990.s004.tif]

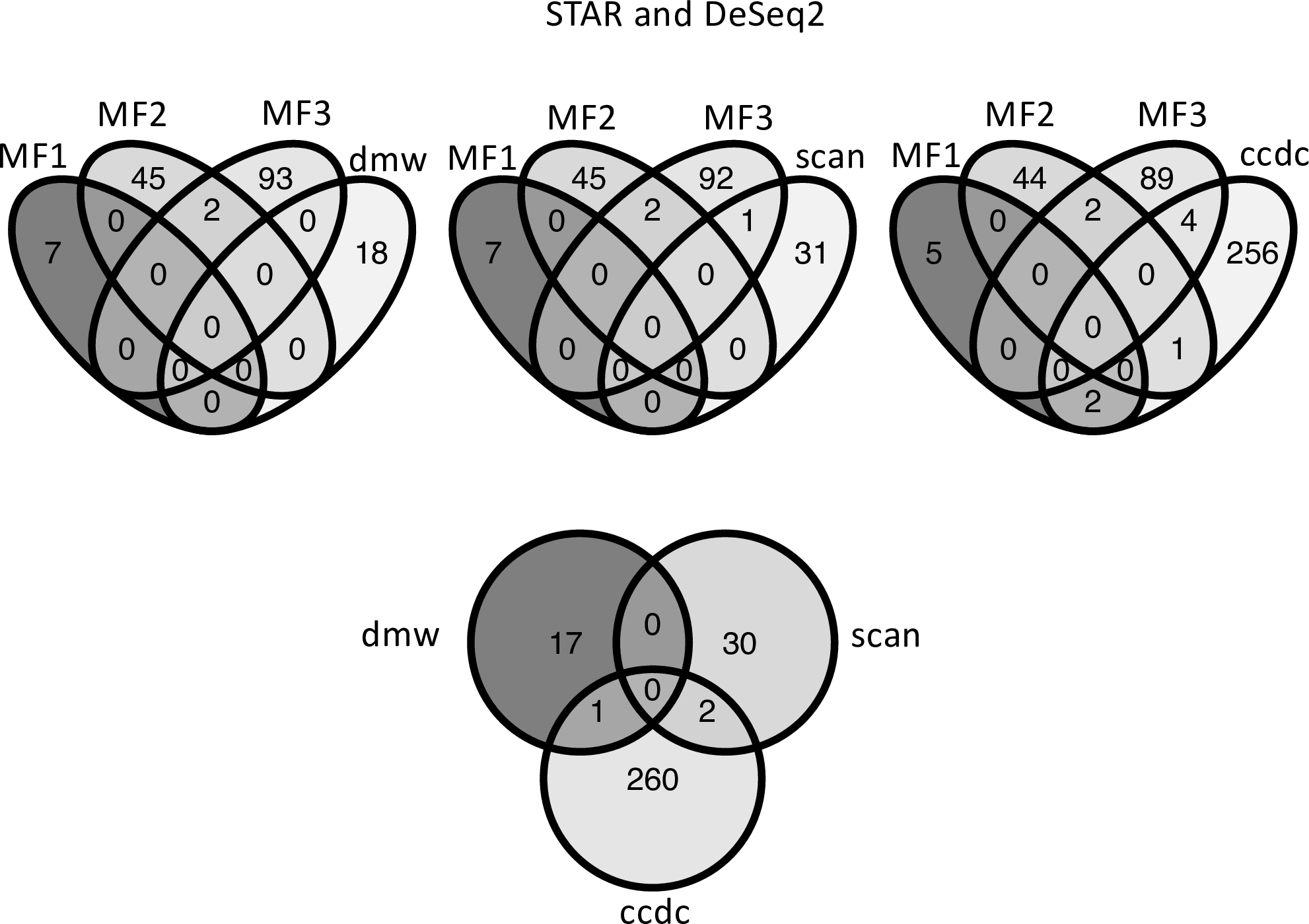

Supplement: S4 Fig — Labeling corresponds with Fig 2. (TIF) [file pgen.1010990.s005.tif]

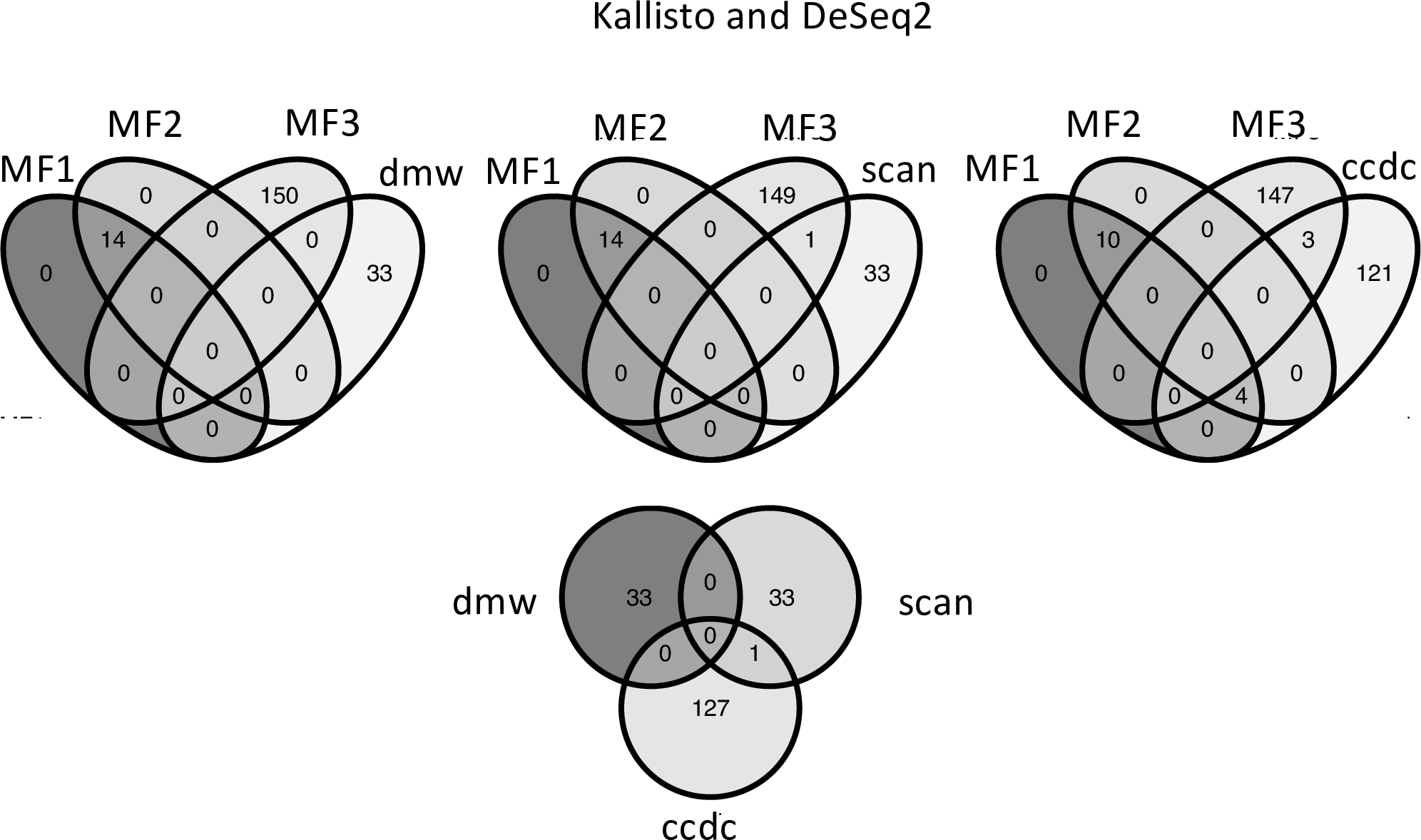

Supplement: S5 Fig — Labeling corresponds with Fig 2. (TIF) [file pgen.1010990.s006.tif]

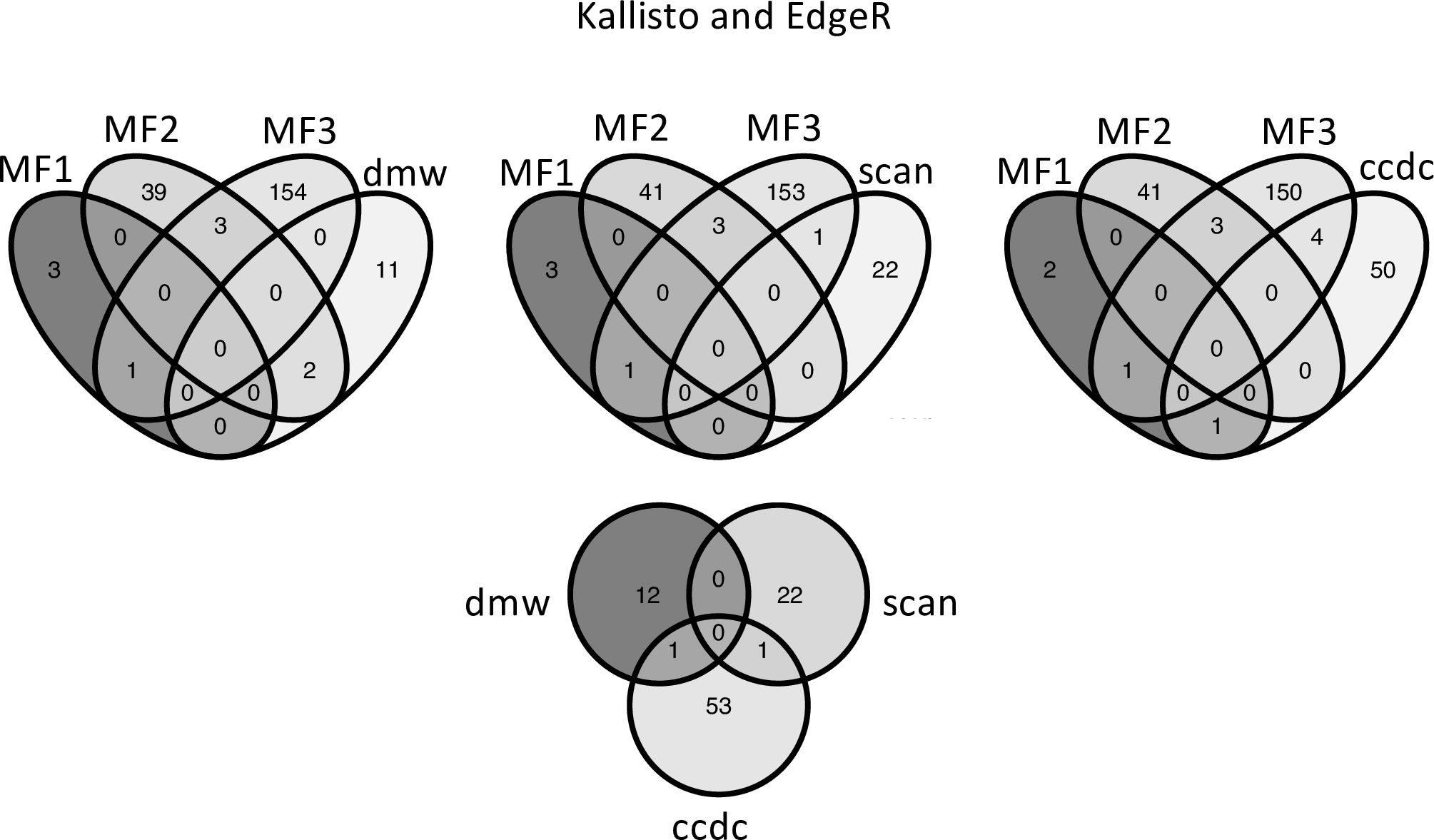

Supplement: S6 Fig — Labeling corresponds with Fig 2. (TIF) [file pgen.1010990.s007.tif]

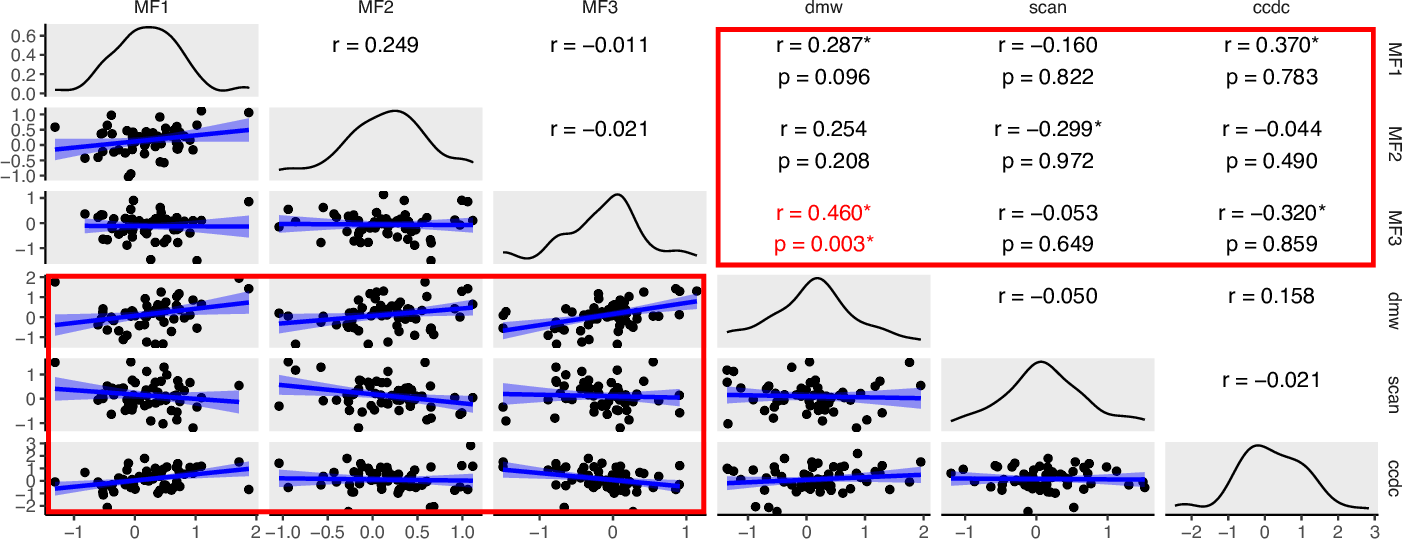

Supplement: S7 Fig — Labeling follows Fig 3. (TIF) [file pgen.1010990.s008.tif]

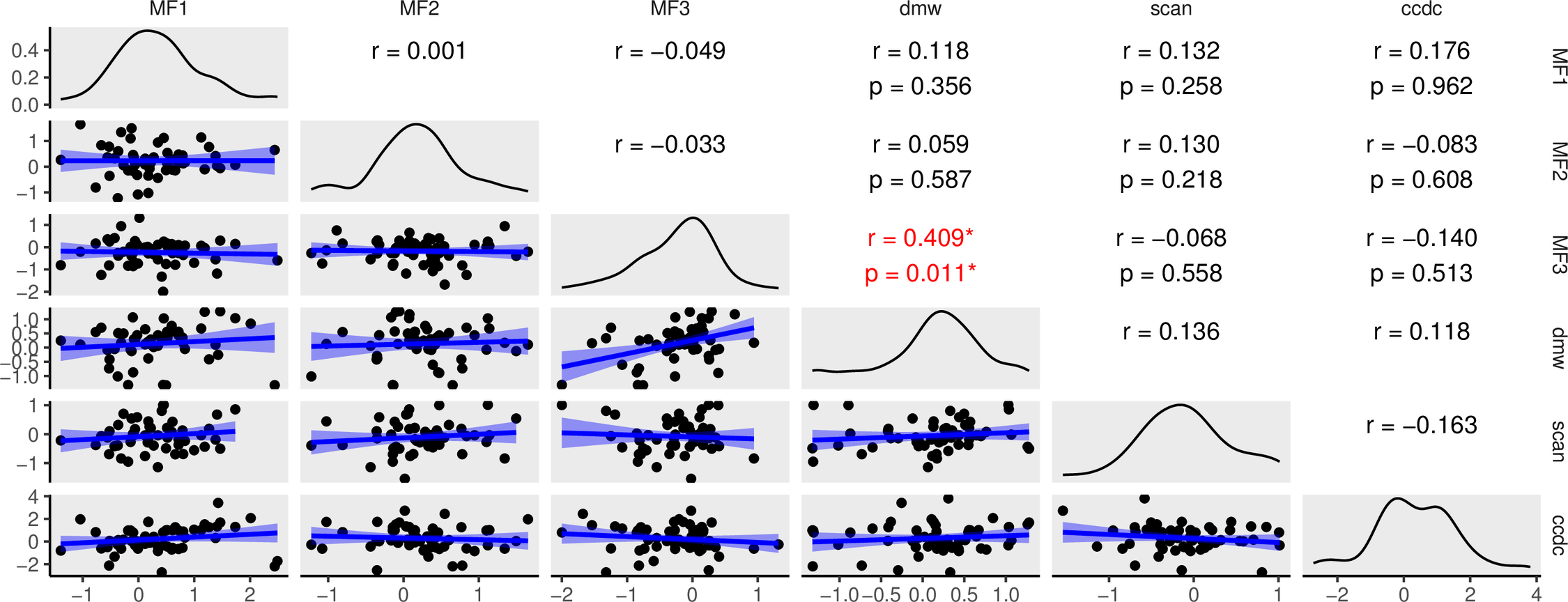

Supplement: S8 Fig — Labeling follows Fig 3. (TIF) [file pgen.1010990.s009.tif]

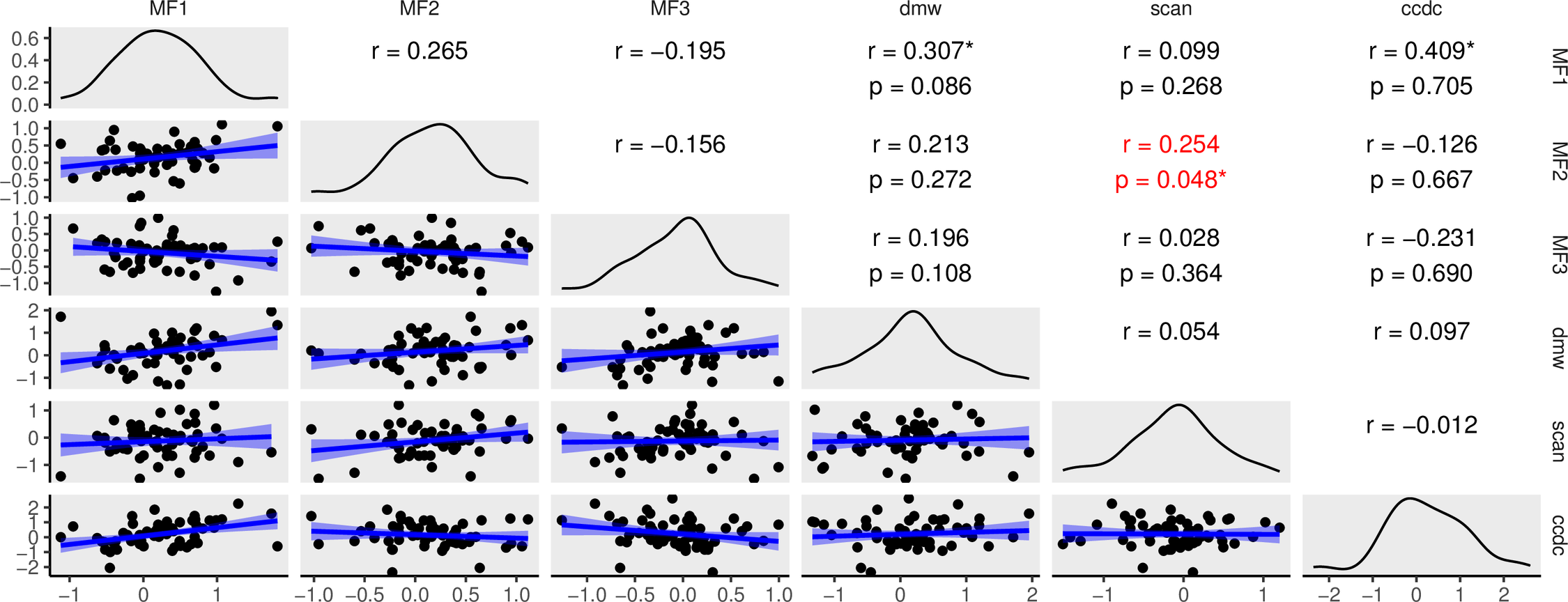

Supplement: S9 Fig — Labeling follows Fig 3. (TIF) [file pgen.1010990.s010.tif]
